# Improving the Utility of Voluntary Ovine Fallen Stock Collection and Laboratory Diagnostic Submission Data for Animal Health Surveillance Purposes: A Development Cycle

**DOI:** 10.3389/fvets.2019.00487

**Published:** 2020-01-24

**Authors:** Sue C. Tongue, Jude I. Eze, Carla Correia-Gomes, Franz Brülisauer, George J. Gunn

**Affiliations:** ^1^Epidemiology Research Unit, Department of Veterinary and Animal Science, Northern Faculty, Scotland's Rural College, Inverness, United Kingdom; ^2^Biomathematics and Statistics Scotland (BioSS), JCMB, Edinburgh, United Kingdom; ^3^SRUC Veterinary Services, Scotland's Rural College, Inverness, United Kingdom

**Keywords:** surveillance, fallen stock, ovine, mortality, syndromic, diagnostic submission, fasciolosis, existing data

## Abstract

There are calls from policy-makers and industry to use existing data sources to contribute to livestock surveillance systems, especially for syndromic surveillance. However, the practical implications of attempting to use such data sources are challenging; development often requires incremental steps in an iterative cycle. In this study the utility of business operational data from a voluntary fallen stock collection service was investigated, to determine if they could be used as a proxy for the mortality experienced by the British sheep population. Retrospectively, Scottish ovine fallen stock collection data (2011–2014) were transformed into meaningful units for analysis, temporal and spatial patterns were described, time-series methods and a temporal aberration detection algorithm applied. Distinct annual and spatial trends plus seasonal patterns were observed in the three age groups investigated. The algorithm produced an alarm at the point of an historic known departure from normal (April 2013) for two age groups, across Scotland as a whole and in specific postcode areas. The analysis was then extended. Initially, to determine if similar methods could be applied to ovine fallen stock collections from England and Wales for the same time period. Additionally, Scottish contemporaneous laboratory diagnostic submission data were analyzed to see if they could provide further insight for interpretation of statistical alarms. Collaboration was required between the primary data holders, those with industry sector knowledge, plus veterinary, epidemiological and statistical expertise, in order to turn data and analytical outcomes into potentially useful information. A number of limitations were identified and recommendations were made as to how some could be addressed in order to facilitate use of these data as surveillance “intelligence.” e.g., improvements to data collection and provision. A recent update of the fallen stock collections data has enabled a longer temporal period to be analyzed, with evidence of changes made in line with the recommendations. Further development will be required before a functional system can be implemented. However, there is potential for use of these data as: a proxy measure for mortality in the sheep population; complementary components in a future surveillance system, and to inform the design of additional surveillance system components.

## Introduction

In the last two decades the surveillance of animal, especially livestock, populations has become an increasingly discussed topic. “Surveillance” has emerged as a discipline in its own right, rather than just another field within the epidemiologist's province (1). Systems, methods, data and designs have been reviewed and evaluated [(2–9), amongst others]. This activity has been driven by a number of parties for a variety of reasons. These include: government officials, who have to mitigate the effects of disease outbreaks and do not wish to be surprised by either the occurrence of specific threats (such as incursions, or outbreaks, of exotic or zoonotic diseases) or by the (re)emergence of known diseases, while operating in an environment; industry bodies, who want to know how much disease is out there, estimate the losses incurred and identify what they can do to improve the health and the productivity of their sector, and researchers themselves, keen to explore new arenas.

The use of automated bio-surveillance systems for outbreak detection and syndromic surveillance (SynS) in the human field (10–14) stimulated studies within the veterinary sphere, reviewed in Dupuy et al. (15). They found that, in 2013, although there were 27 veterinary syndromic surveillance systems in 12 European countries, most of these did not yet have the statistical wherewithal to adequately analyse the data. Subsequently, there have been additional studies [e.g., (16–22)] and increased momentum within the animal health surveillance sector. Although there is evidence of further development of digital surveillance systems for animal populations (23), they have not yet matured into fully functional, digital, automated bio-surveillance systems providing outbreak detection, syndromic surveillance, monitoring of trends and situational awareness in animal populations.

A major constraint is the lack of computerized, automatically collected data (2). This apparent scarcity may be driven more by a lack of accessibility and availability, plus issues of data management (e.g., poor data quality, absence of core data needed for analysis), design (e.g., coverage) and documentation rather than an absence of data sources (9, 24, 25). However, in the United Kingdom (UK), there are continued demands to make use of available health-related information, from alternative existing data sources that were not designed for surveillance purposes, as surveillance intelligence within a wider surveillance system (26, 27). Elsewhere the potential to use national, or regional, statutory, or mandatory, centralized registers of cattle, pig, or equine identification, movements and fallen stock to provide measures of excess mortality has been investigated (28–35). The attractiveness of these registers arises from the tranche of European legislative requirements to record births, movements and deaths of individual animals and to dispose of livestock that die without passing through slaughterhouses, by appropriate means (https://eur-lex.europa.eu/). The existence of single systems for registration, or centralized collation of data from a number of rendering plants, or collection centers, facilitates this; although analyses can still be challenging and careful interpretation is required. These challenges are compounded when recording of death is not necessarily at an individual animal level, when there is no centralized collation of electronic data, and when a free market system operates i.e., farmers can choose from multiple fallen stock collection providers, who themselves can chose the level of service provision. This is the case with the British fallen stock collection system, especially for the ovine population. Attempts to use such data result in yet another “particularly interesting situation for epidemiologists,” despite this being a small, rather than Big Data, scenario (36).

The overall aim of these studies was to assess whether data from a fallen stock scheme with voluntary membership had potential for use for livestock health and disease surveillance purposes.

The initial objectives of this study were: firstly, to describe the mortality experienced by British sheep, as measured by collection of ovine material from National Fallen Stock Company (NFSCo) members; secondly, to determine if there were any temporal (i.e., seasonal or annual) trends and/or spatial clusters associated with mortality and thirdly, to investigate the application of an aberration detection algorithm. The focus of the study was the utility of the data, rather than the statistical methods i.e., Could these objectives be achieved when the data collection was not designed with these purposes in mind? In the process, any limitations of the data would be identified, as would ways that they might be addressed. Analysis of the initial available dataset raised additional questions of relevance to the development of a functional surveillance system component. One of these was whether an additional existing data source—laboratory diagnostic submissions data collected as part of a passive surveillance system—could be utilized in parallel to the fallen stock collections data, in order to provide insight and facilitate interpretation of any statistical alarms that might be raised in the latter. The study was therefore extended with the addition of an objective to explore the utility of these data, using an exemplar diagnosis. After all, the definition of animal health surveillance implicitly includes action, or at least the inclusion of an action plan, with regard to the implementation of interventions that aim to mitigate the identified risk (37, 38).

## Materials and Methods

There were four developmental stages. These were: Stage 1—an exploration of the utility of 2011–2014 Scottish ovine fallen stock data, leading to initial results and initial recommendations; Stage 2—extension of the analyses to 2011–2014 England and Wales ovine fallen stock data, leading to initial results and further recommendations; Stage 3—an exploration of the utility of 2011–2014 Scottish ovine fasciolosis diagnostic data, specifically to see whether it could contribute to interpretation of the Stage 1 outputs; Stage 4—an update using 2015- mid-2018 Scottish plus England and Wales ovine fallen stock data, leading to updated results due to increased interest being shown by third parties.

### Available Data

#### Fallen Stock Collection in Great Britain and the National Fallen Stock Company (NFSCo)

In accordance with EU Animal By-Product regulations (39), livestock that dies on British farms must be collected, identified and transported away as soon as is reasonably practical, by an approved transporter. Disposal must be via an approved, registered animal by-products premise (40). Exceptions are made for pet animals and horses, remote areas including large parts of the Scottish Highlands and Islands (41) and in cases of natural disaster. Livestock keepers can make arrangements themselves directly with one of the many companies that deal with collection and disposal, or the process can be facilitated by becoming a member of the National Fallen Stock Company (NFSCo) (40). A collection service or a disposal premise may operate privately and/or as a member of NFSCo.

NFSCo CIC is a not for profit Community Interest Company that acts as an intermediary between the farming community and fallen stock collectors across Great Britain (GB) (www.nfsco.co.uk). It provides a service; sets standards; promotes high levels of bio-security; creates competition; facilitates payments and simplifies invoicing and cash flow. Membership is voluntary and free. Data are, therefore, collected for business needs. When a collection is made from a members' property, receipts are provided for monthly invoicing purposes. The number of Species Service Units (SSUs) that are collected on date X, from postcode Y, belonging to member Z, are recorded.

The definition of “Fallen Stock” for NFSCo is “animals which were killed (euthanasia with or without definite diagnosis) or have died (including stillborn and unborn animals) on farm and which were not slaughtered for human consumption. This includes animals killed by routine culling as part of normal production arrangements, where no government support is applied and animals lost during events that would ordinarily be covered by existing insurance arrangements e.g., fires and road accidents.” (www.nfsco.co.uk). NFSCo collections are, therefore, an indication of the mortality experienced in the livestock populations that belong to NFSCo Members.

#### British Postcodes

A full British postcode consists of two parts of up to eight specific alpha-numeric characters (i.e., **AA**00 00BB). The first two alphabetic characters define a postcode area e.g., IV—Inverness, CH—Cheshire. There are 124 postcode areas in the United Kingdom of Great Britain and Northern Ireland (UK); they do not equate to country boundaries. The unit code (i.e., the last two letters, AA00 00**BB**) part of the full postcode identifies a postal route (or walk) of up to 100 addresses, for delivery purposes. A property may, therefore, have its own unique full postcode, or there may be multiple properties per full postcode (42). Thus, in an area of high livestock holding density, or where the area that the unit code applies to is large, more than one collection point may have the same postcode. They may, or may not, belong to different members and members might have multiple collection points.

#### Available NFSCo Datasets

Stages 1 and 2: Separately for each of the three countries, Scotland, England and Wales, two datasets were provided: a membership file and a Species Services file for sheep.

Stage 4: Two Species Services files for sheep were provided; one for Scotland and one for England and Wales together.

##### Membership files

These files provide an anonymized snapshot of the entire NFSCo membership, for each of the three countries, as of the date that the file was extracted. For Scotland this was “as of 31st October 2014,” whereas for England and for Wales it was “as of 31 July 2015.” Each member record had a unique identifier, a user status and postcodes for each of the addresses belonging to that member. There were no join, start, or stop dates and no other attribute data such as livestock species belonging to the member, livestock numbers, management type, or species collected from a member.

##### Species service file—sheep

The sheep Species Services files contain aggregated Species Service Unit data (SSU).

Types of SSU: In the Stages 1 and 2 files up to 11 types of SSU were recorded, although not all types were present in every country's file (Table 1). In Stage 4, there were a small number of additional SSUs, mainly in the England and Wales dataset. These mainly occurred infrequently apart from Dolav® sheep per kilo. This refers to a type of industrial plastic pallet box that can be used to store material.

**Table 1:** The 11 original SSUs recorded in the Sheep services files and what they consist of.

**Species service unit**	**Explanation 1 unit equals…**
Lambs 0–1 month^c^	1 unit = 1 lamb, between birth and 1 month of age
Lambs 2–12 month^c^	1 lamb, between and including 2–12 months of age
Sheep over 12 month^c^	1 sheep, over 12 months of age
10 + bags of lambs^c^	10 or more bags of lambs, under 12 months of age
10 + Lambs 2–12 month^c^	10 or more lambs, between and including 2–12 months of age
10 + Sheep over 12 month^c^	10 or more sheep, over 12 months of age
Sheep/lambs per 10 kg^c^	10 kg of dead ovine
Container/bag for lamb^c^	one container or bag for putting lambs in
10 + lambs 0–1 month^a^	10 or more lambs, between and including 0–1 months of age
Sheep/lambs per 10LTR^b^	One 10 liter volume unit of mixed ovine material
Unweighed Skip – Sheep^b^	One unspecified size skip of adult ovine material

a In English data files only;

b In English and Welsh data files only;

c *In all three data files*.

Time periods and aggregation:

In the original datasets (Stages 1 and 2), the time period available was January 2011 to December 2014, inclusive, for Scotland and January 2011 to July 2015 inclusive, for each of England and Wales. Only the period to the end of December 2014 was analyzed initially. SSUs were aggregated by full postcode and by calendar month. This level of aggregation was defined by the data providers.

In Stage 4, the time period provided was January 2015 to June 2018, inclusive, for both the Scotland and combined England and Wales datasets. SSUs were aggregated by a unique ID representing the membership number, full postcode and by date of collection. These data were combined with the Stages 1 and 2 data to produce a dataset for the full period 2011 to mid-2018 for Scotland and for England and Wales.

#### SRUC Veterinary Services Fasciolosis Data (Stage 3)

The SRUC Veterinary Services (VS) Disease Surveillance network consists of eight centers (DSCs) located around Scotland. They receive diagnostic submissions from livestock keepers, in conjunction with their veterinarians. These submissions are made to confirm, or elucidate, a diagnosis in cases where diagnostic uncertainty exists based on clinical examination. A submission may consist of single or multiple samples from single, or multiple animals. All submissions are recorded in a laboratory information management system (LIMS). For diagnostic samples, when a diagnosis is reached according to specific criteria then a standardized Veterinary Investigation Diagnosis Analysis (VIDA) code is applied to that submission. There are two fields in which such a diagnosis can be recorded (VIDA1 and VIDA2). These may refer to a primary and secondary diagnosis in the same animal, or to two primary diagnoses in two different animals included in the same submission.

Acute fasciolosis is often fatal in sheep (43). For exploration in parallel with the descriptive spatio-temporal analysis of fallen stock data, the interest in fasciolosis diagnoses is as a potential explanatory factor for statistical alarms indicating increased mortality. For sheep, the most appropriate submissions should be those diagnosed as VIDA CODE 372 Acute fasciolosis (VIDA372). Chronic fasciolosis is recorded as VIDA CODE 373 (VIDA373) (Table SM1).

All ovine diagnostic submission records between January 2011 and December 2014 inclusive that had VIDA372 or VIDA373 recorded as a diagnosis were extracted from LIMS. These submissions consisted of carcase, liver, and/or feces samples appropriate to the VIDA CODE assigned (Table SM1).

### Data Transformations

#### Assumptions Made in Order to Convert SSUs to Animal Units (AUs)

The values for the SSUs “container/bag for lamb” were excluded as they record a charge for the provision of containers, not for ovine material collected.

There are three age groups of interest: lambs from 0 to 1 month of age; lambs from 2 to 12 months of age, and sheep over 12 months of age. Some assumptions were needed to convert the SSUs in the datasets into animal units (AUs). For the SSU 10 + [age group], an estimate of the AUs collected can be made by multiplying the number of these SSUs by 10. This will be a consistent under-estimate, as this is the minimum number for this SSU. It was assumed, based on the frequency distributions over time of SSU collections and on industry knowledge, that bags of lambs would be most likely to consist of lambs between 0 and 1 months of age, usually in lambing periods and that there would be a minimum of four lambs per bag, The number of collections of 10+ bags of lambs was thus multiplied by 40, to give an estimate of the number of 0–1 month lamb animals units that they represented.

The unit by weight (Sheep/lambs per 10 kg) occurred as integers (i.e., 1, 2, 3 etc.), with each unit equating to 10 kg of dead ovine material. More than half of the collections were five units (50 kg) or less with the peak period contemporaneous with peaks for numbers of Lambs 0–1 month and co-incident with the peak period for numbers of Sheep over 12 months (data not shown). It was assumed that, firstly, an average weight for a dead lamb would be 5 kg and, secondly, the weight of a dead sheep over 12 months of age is likely to be similar to, or lower than average breed live-weights (kg). As the latter vary considerably depending on breed, it was assumed that a minimum weight for a sheep over 12 months of age would be more than 45 kg. These assumptions led to formulae for conversion of by 10 kg collections into AUs (Table 2).

**Table 2:** Relationship between Sheep/lambs per 10 kg and animal units (AUs) in Stages 1, 2, and 4.

**Species service unit**	**Number of animal units**
Sheep/lambs per 10 kg— <5 units	Less than 50 kg of dead ovine = number of units*10 = kg At 5 kg a lamb = Kg/5 = number of 0–1 month lambs
Sheep/lambs per 10 kg—5 or 6 units	Number of units*10 = kg = 50–60 kg Count as 1 sheep over 12 months
Sheep/lambs per 10 kg—7 or 8 units	Number of units*10 = kg = 70–80 kg Treat as a mixture—one adult sheep of 45 kg and the rest is made up 0–1 month lambs so the remaining weight in kg/5 = number of lambs of 0–1 month
Sheep/lambs per 10 kg—9 to 12 units	Number of units*10 = kg = 90–120 kg Count as 2 sheep over 12 months
Sheep/lambs per 10 kg—more than 12 units	Number of units*10 = kg = > 120 kg Kg/45 = number of sheep over 12 months—the rest is made up 0–1 month lambs so the remaining weight in kg/5 = number of lambs of 0–1 month

Records for Sheep/lambs per 10LTR collections and for Unweighed Skip—Sheep collections were rare. Records for Dolav® sheep per kilo started in 2015 and were more frequent, although the approximate numbers of collections annually were consistent for England and Wales for the period 2015–2018. No clear assumptions could be made as to what these SSUs represented in terms of AUs. They were therefore excluded from further analysis.

##### Validation of assumptions for conversion of fallen stock data to animal units

The assumptions were tested for the Scottish data analyses, by consultation with a Sheep Research-Industry Interface Group and a Veterinary Advisory group.

#### SRUC VS Submissions Data

The VS submission records were aggregated into postcode areas from the full postcode provided and into calendar month from the submission date (day/month/year), in order to be comparable with the fallen stock data.

Only two of the age groups in the fallen stock data, lambs of 2–12 months of age and sheep over 12 months of age, are of relevance for fasciolosis due to the life cycle of the liver fluke. Additional data fields were used to categorize the submissions into the relevant age groups.

A VIDA diagnosis (i.e., submission records, hereafter also referred to as a “case”) was used as one unit of analysis. However, although a diagnosis is applied at submission level, a submission may consist of single or multiple samples from single, or multiple animals. Additional data fields were used, therefore, to attempt a conversion to AUs. This was used as a separate unit of analysis. Where data were missing an assumption of 1 AU for each submission was made.

### Analytical Methods

#### Spatial Aggregation

Country and postcode area were the spatial units of analysis used for the Scottish data. While country was used for both England and Wales in Stage 2 and England and Wales combined in Stage 4, postcode area was not feasible for use in England and Wales. This was due to a combination of factors. Postcode areas were therefore aggregated into larger spatial areas across both England and Wales, as regional units specifically for the purposes of these analyses. These regions were given descriptive assigned names that do not directly correlate with other regional classification systems. In Stage 3, the VS data were analyzed at country level only.

#### Descriptive Summaries

##### Fallen stock membership

Stages 1 and 2 membership data were briefly described on a country basis to provide information on the levels of membership, duplication of collection points per member and per postcode area (Scotland) or by new regional areas (combined England and Wales).

##### Fallen stock species service and animal units

In Stages 1 and 2 only, the number of units of each of the SSUs collected, per month, was plotted over the studied time period. This assisted in determining the assumptions for conversion into AUs, described above.

In Stages 1 and 2, to examine whether or not the effect of omission and the potential for mis-classification (when converting SSUs to AUs) made a substantial alteration to the outputs all AU analyses were run without any of the multiple SSUs included and without the ovine material (kg) contributions and then the outputs were compared. With each of the multiple units and ovine material (kg) contributions included sequentially, the new estimates of AU (i.e., re-categorized variables) were then analyzed and compared. Consequently, the variables that included the estimated AU from both multiple units and the kg conversion were used for each of the three age groups: All lambs 0–1 month; All lambs 2–12 months; All sheep over 12 months. These are the outputs reported in this manuscript and they were the AU categories that were used for each age group in Stage 4 (January 2011 to June 2018 inclusive).

Stages 1, 2 and 4: The number of AUs were summarized and described: per age group, by country, by postcode area or regional area, by year and by month and combinations thereof.

##### SRUC VS fasciolosis data

Stage 3: The number of VIDA372 (acute fasciolosis) and VIDA373 (chronic fasciolosis) diagnoses, in terms of submissions assigned a diagnosis (cases) and as estimated AUs in those submissions, were summarized and described for the country as a whole, and by postcode area, by age group and by time in a manner comparable to that used for the fallen stock data.

#### Time-Series Analysis

Descriptive time-series analyses were used to investigate and visualize temporal patterns in all of the datasets (fallen stock and VS). The latter was restricted to only the diagnostic submission records of Scottish origin with primary fasciolosis acute fluke (VIDA372) diagnoses.

The monthly time-series, seasonal pattern, annual totals, and average annual trend were plotted. Seasonal patterns were represented as monthly boxplots, to depict both the seasonal pattern and reflect differences in the average monthly number of events/units—where an event is the relevant unit of analysis—and variations across months. The monthly seasonal effects (indices) were then computed by, firstly, eliminating the trend from the data by dividing each monthly observation by their yearly average. This quotient is then averaged for each month across all the years. This gives an indication of how the value in a particular month differs from what is expected per month on average. The average annual trends were plots of the average yearly events, where average could be either mean or median number of events in a given year. The default was usually the mean; the median was used for data with strong outliers in order to minimize the influence of the outliers on the shape of general trend. These analyses were performed at country level for each stage, by age group.

For the fallen stock data, time-series plots were also plotted for each postcode area (Scotland) and region (England and Wales), by age group. Differences in average annual trends, seasonal patterns and variations across these spatial areas were captured in these plots. These area time-series data were later used to fit temporal alarm detection algorithms (TADA).

#### Aberration Detection

The datasets were scanned using the classic (original) Farrington method (44) that allows for analysis of counts without adjustments. Data were modeled as over-dispersed Poisson counts. A subset of the available data was used as a reference period to inform the models and account for seasonality. The 12 months of the first year (2011) were used in Stages 1, 2, and 3. The generalized linear models were used to predict what would be expected to occur i.e., to construct an upper prediction interval, or threshold, at 0.01 level of uncertainty. These threshold values are compared with the observed value at each time point from January 2012 to December 2014. If the actual value exceeds the threshold, an alarm is raised. The same algorithm was used for analyses of Stage 4 data but the first 24 months (2011 and 2012) were used as the reference period values to reflect the longer time-series and to improve the model performance.

All analyses were done in R statistical package (45) and the TADA was fitted using the surveillance package (46).

## Results

### Data

#### Lack of Denominator Data

There were no suitable denominator data available for either the fallen stock or the VS data. Due to lack of mutual farm or premise identifiers, these two data sources could not be linked and no alternative data sources could be linked with the fallen stock datasets to augment them and provide a denominator dataset, or additional attribute data. Therefore, subsequent analysis had to use count data.

#### Spatial Aggregation

**Figure 1:**
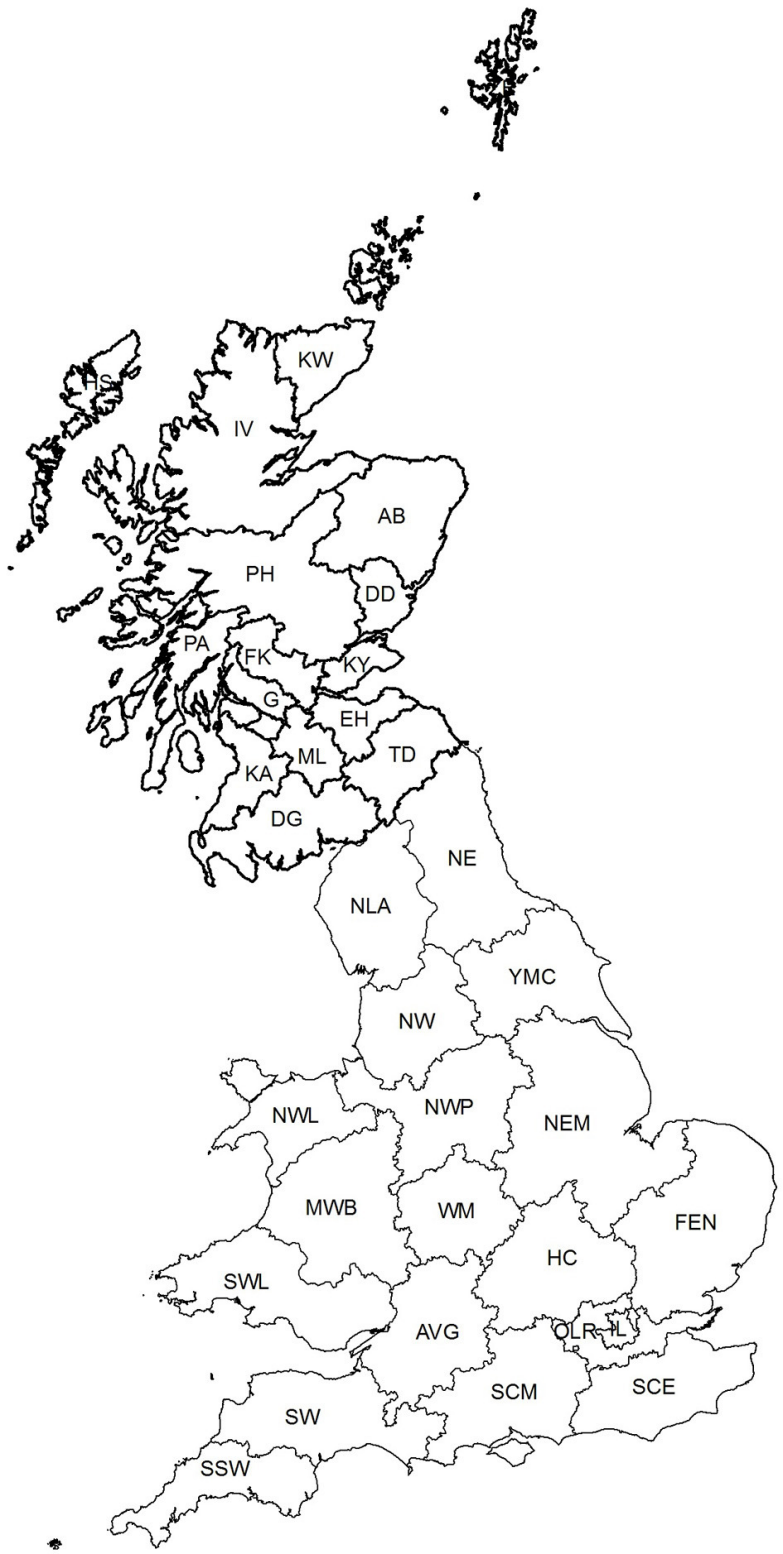
The location of the aggregated spatial units used in the analyses−16 postcode areas in Scotland and 18 regional areas in England and Wales (see Tables SM2, SM3 for details). Ordnance Survey data © Crown copyright and database right 2017; Royal Mail data © Royal Mail copyright and database right 2017 and National Statistics data © Crown copyright and database right 2017.

Stages 1 and 4: Fourteen mainland Scottish postcode areas were represented in the fallen stock collections data (Figure 1 and Table SM2). An additional one, the Outer Hebrides, was present in the Scottish membership file.

Stage 2: There were more than 90 postcode areas in the English membership file and nine in the Welsh one. Four of the postcode areas in the English file also appeared in the Welsh file because these postcode areas extend across the border. When aggregated into larger units across both England and Wales, there were 18 designated regional areas (Figure 1 and Table SM3).

Stage 3: Acute fasciolosis cases came from 13 of the 14 mainland Scottish postcode areas, with no diagnoses from Dundee, nor from the Outer Hebrides or Shetland island postcode areas. All 14 of the mainland Scottish postcode areas had at least one chronic fasciolosis case, as did the two additional island areas, Outer Hebrides and Shetland.

### Available Datasets—Analysis

Not all of the background data and figures from which the following results are drawn are included in this manuscript. They are available on request from the corresponding author.

#### Fallen Stock Membership

Numbers of members were similar in Scotland and Wales, with approximately three times as many in England. The majority (90–96%) of active members had only one collection point (CP); however a small proportion (0.06–0.1%) had more than 10 collection points. In some full postcodes, there were multiple active CPs, with a maximum of 10. In Scotland, more than one in five of the full postcodes that contained active CPs, had at least two located within them. This was similar in Wales, whereas in England it was less, at just over one in 10. The spatial distribution of members roughly approximated to expectations, given livestock holding density and size/shape of postcode or regional areas, except for the Outer Hebrides where representation was low.

#### Fallen Stock Animal Units (AU)

The total number of AUs collected each year, stratified by age group varies (Table 3).

**Table 3:** The estimated total number of (all) animal units (AUs) collected from each country by year and age group.

**Animal units**		**Year**							
Country	Age group	2011	2012	2013	2014	2015	2016	2017	2018 (Jan–June only)
Scotland	Lambs 0–1 month	15,436	14,547	18,977	8,980	27,150	31,183	28,210	40,001
	Sheep over 12 months	78,066	64,565	96,891	60,724	65,080	74,120	82,378	94,737
	Lambs 2–12 months	32,678	26,741	30,506	21,096	22,170	29,153	38,092	23,264
England and Wales	Lambs 0–1 month	45,423	48,494	74,059	48,747	32,042	28,229	27,268	29,123
	Sheep over 12 months	115,327	119,722	142,126	118,313	124,962	12,7961	134,585	102,461
	Lambs 2–12 months	27,367	33,683	37,789	32,533	30,959	36,709	39,630	17,870

##### Stage 1—Scotland 2011–2014

In the subset of analyses that built up to the re-categorized “all animal” unit variables, for each of the three age groups: Lambs 0–1 month; Lambs 2–12 months; Sheep over 12 months, no alarms were raised when only the single AUs were used. As components were added, there were slight effects on the shape of the seasonal patterns and the aberrations detected, with more marked effects on the magnitude of the annual trends.

**Figure 2:**
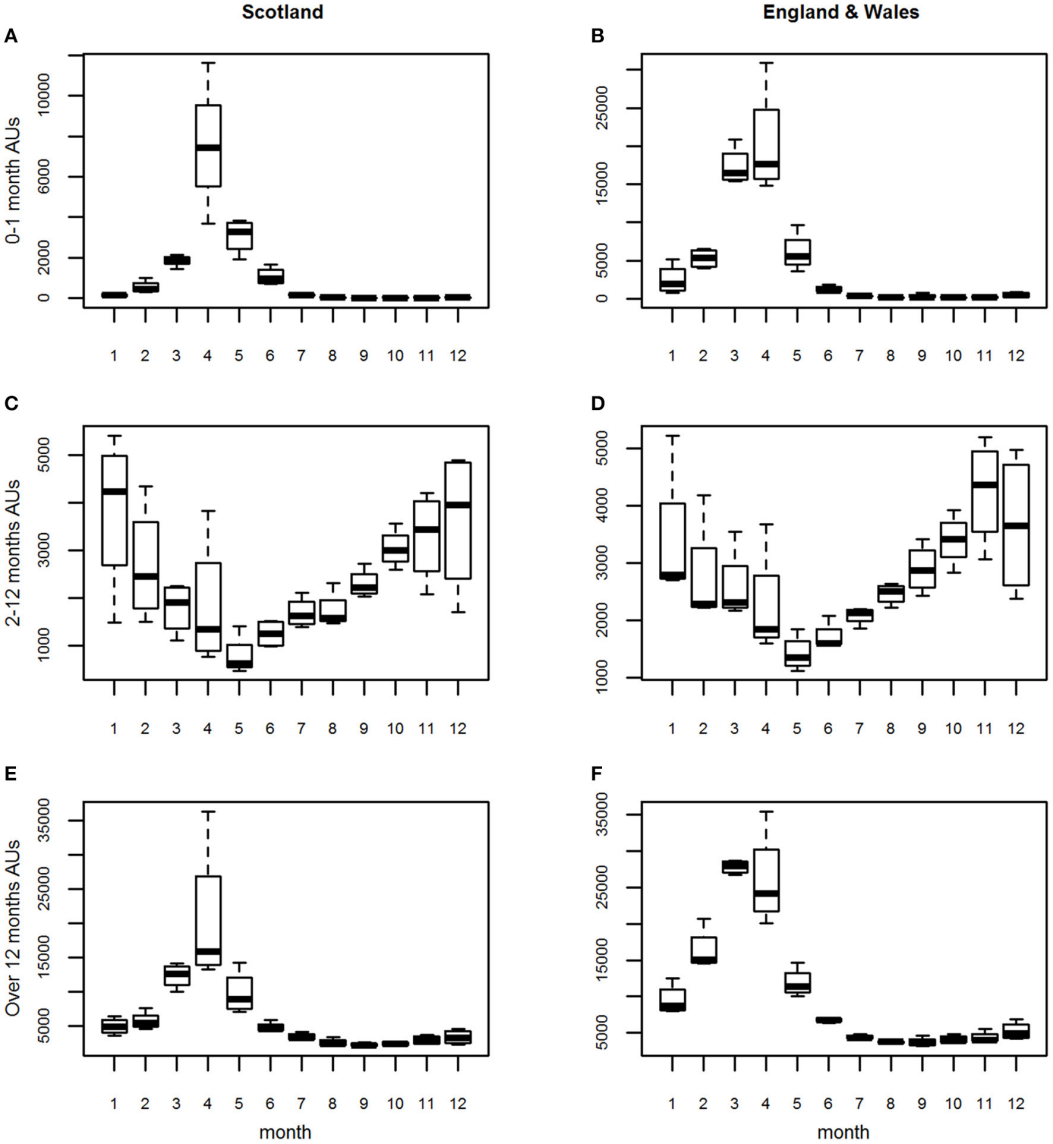
**(A–F)** The seasonal patterns (i.e., distribution of the monthly counts) for the three age groups of estimated animal units (AUs) by country using the 2011–2014 fallen stock datasets.

**Figure 3:**
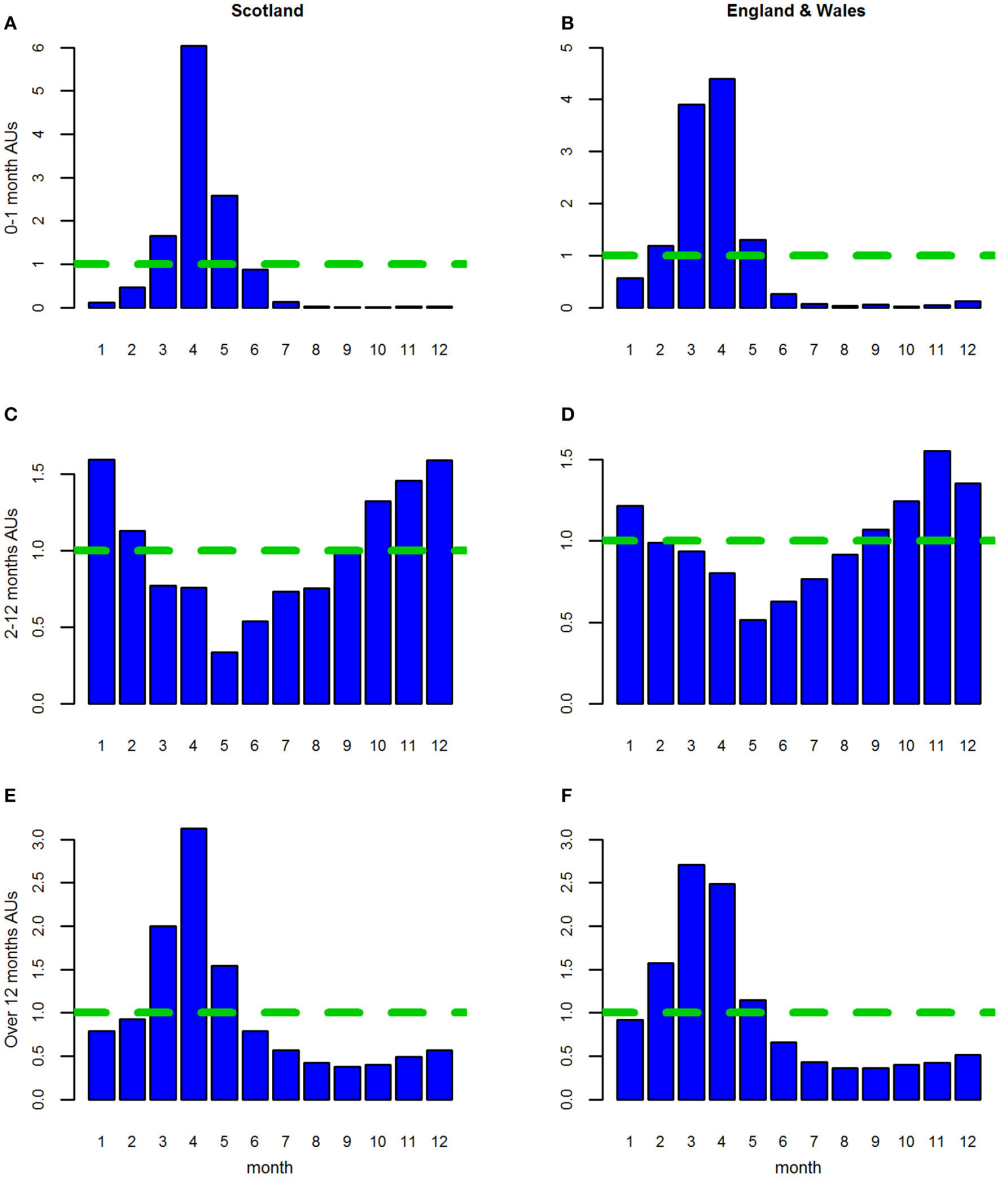
**(A–F)** The seasonal effects for the three age groups of estimated animal units (AUs) by country using the 2011–2014 fallen stock datasets.

For the estimated “all AU” variables, in all three age groups, seasonal patterns with an annual cycle were seen. These were similar for Lambs 0–1 month and Sheep over 12 months but differed for Lambs 2–12 months. This similarity was reflected in the seasonal patterns (Figures 2A,C,E) and seasonal effects (Figures 3A,C,E).

For both Lambs 0–1 month and Sheep over 12 months, the seasonal pattern peaks in April and is at its lowest in August and September, respectively (Figures 2A,E). The seasonal effects are above 1 in the period March to May, with the effect in March higher than in May for Sheep over 12 months and vice versa for Lambs 0–1 month (Figures 3A,E).

The seasonal effect in April is ~5 times the expected monthly average mortality for Lambs 0–1 month and ~2.7 times that for sheep over 12 months. However, the seasonal patterns and effects for Lambs 2–12 months are very different (Figures 2C, 3C). These were at their lowest in May and highest in the late autumn/winter months October to January.

**Figure 4:**
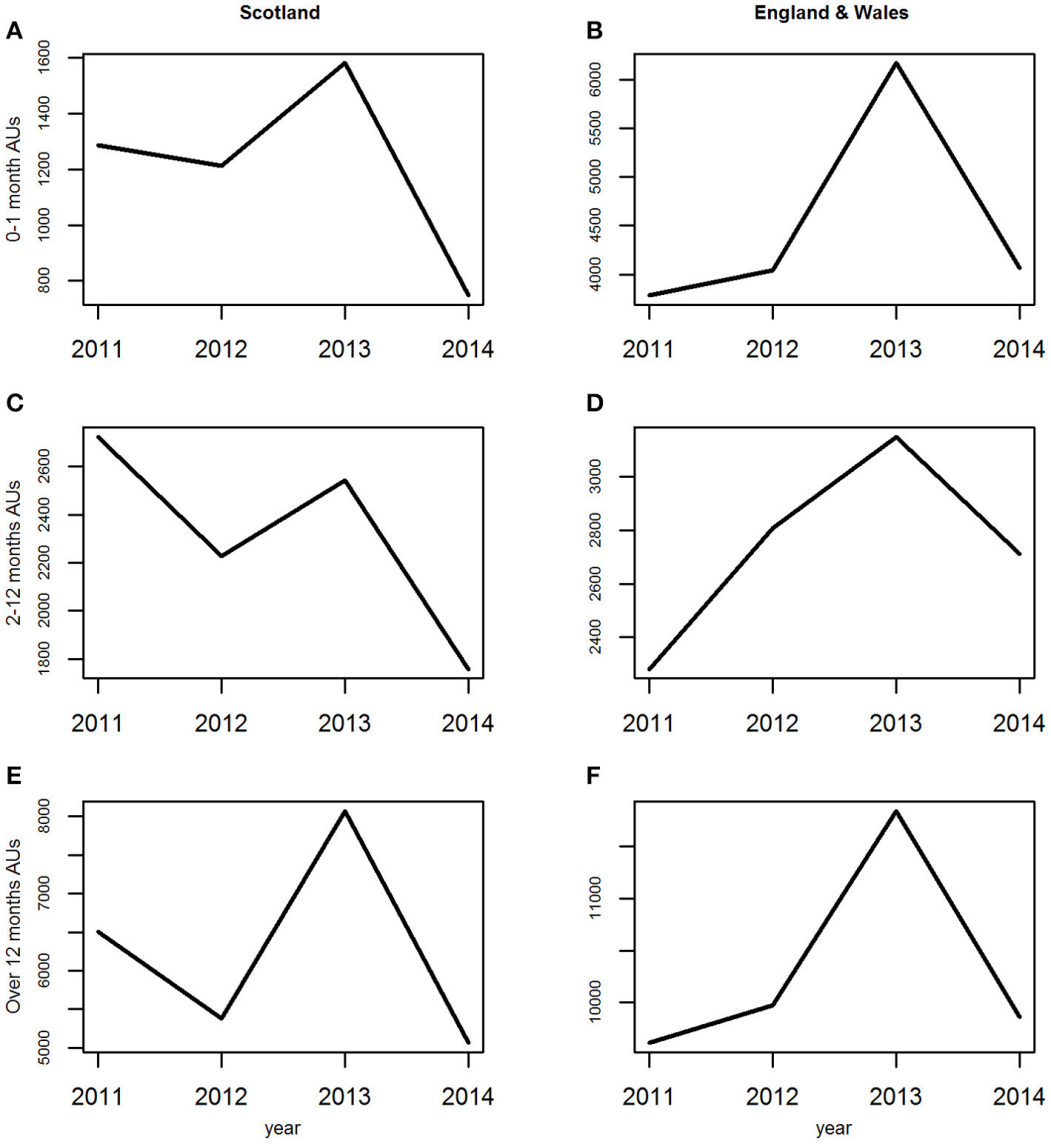
**(A–F)** The average annual trends for the three age groups of estimated animal units (AUs) by country using the 2011–2014 fallen stock datasets.

The average annual trend was similar for all three age groups although the magnitudes of the peaks and troughs differed slightly (Figures 4A–F). These dropped sharply from 2011 to 2012, rose in 2013 and dropped again in 2014.

**Figure 5:**
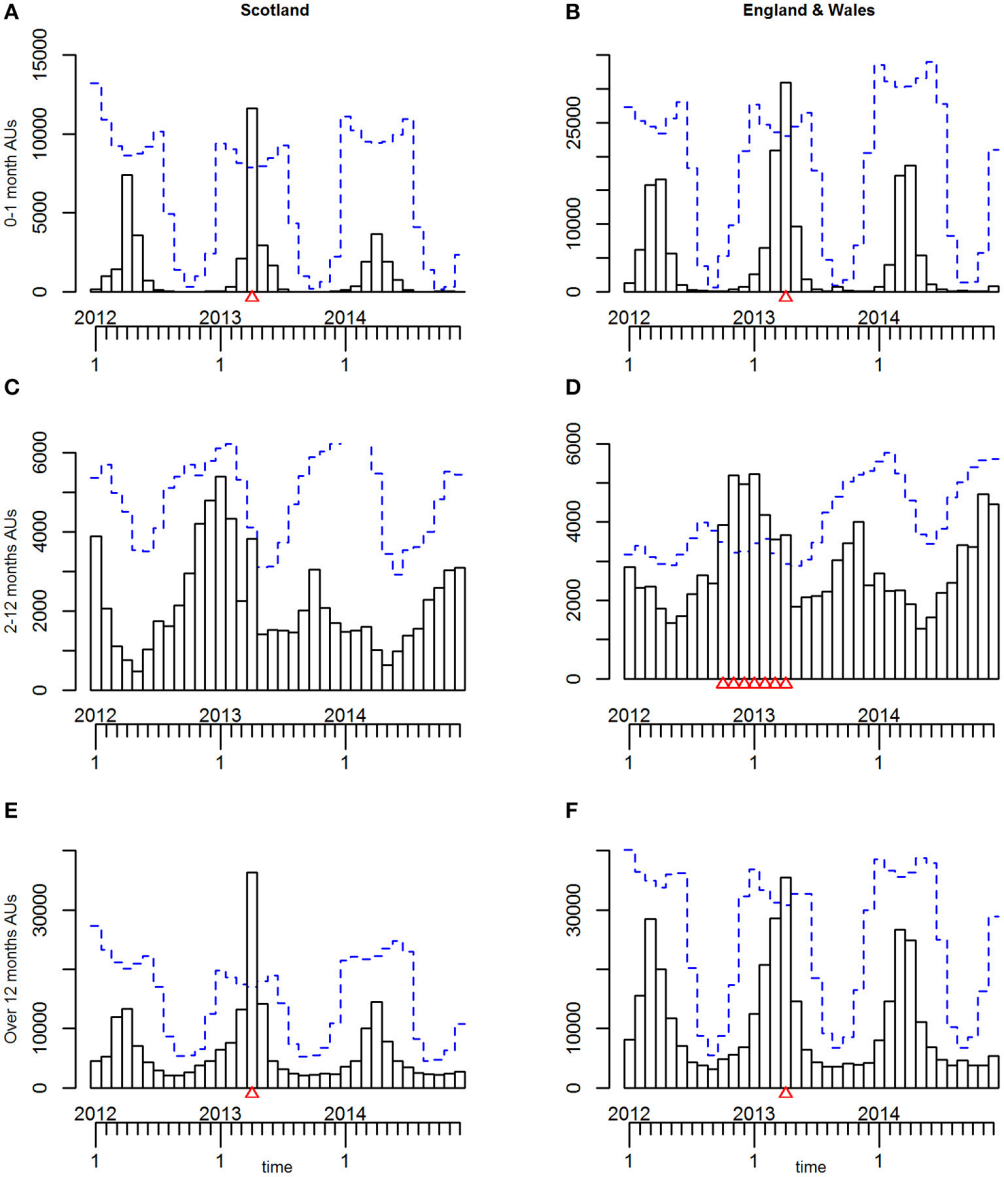
**(A–F)** The time-series plots and temporal aberration detection (TADA) for the three age groups of estimated animal units (AUs) by country, using the 2011–2014 fallen stock datasets, with 2011 as the reference period.

At a country level, the predicted threshold for the TADA was exceeded and an alarm triggered in April 2013 for both Lambs 0–1 month and Sheep over 12 months (Figures 5A,E and Table 4), whereas no alarm was triggered throughout the study period for the Lambs 2–12 months (Figure 5C and Table 4).

**Table 4:** Time-points at which country level statistical alarms were raised by the application of the Farrington TADA to the datasets, by country, dataset and age group.

		**Age category**		
**Time period**	**Country area—dataset**	**Lambs 0–1 month**	**Sheep over 12 months**	**Lambs 2–12 month**
2011–2014 reference = 2011	Scotland—fallen stock (Figures 5A,C,E, respectively)	April 2013	April 2013	None
	England—fallen stock		October 2012	October 2012 to
		April 2013		April 2013 inclusive bar March 2013
		September 2013		
	Wales—fallen stock	October 2012	October 2012	October 2012 to April 2013 inclusive
		March 2013	April 2013	September 2013
	England and Wales combined—fallen stock (Figures 5B,D,F, respectively)		October 2012	October 2012 to
		April 2013	April 2013	April 2013 inclusive bar March 2013
		September 2013		
	Scotland—VIDA372 Animal Units	n/a	January 2012	January 2012
			March 2012	
			October 2012 to February 2013 inclusive	October 2012 to January 2013 inclusive
			May 2013	
	Scotland—VIDA372 Submission-level assigned diagnosis units	n/a	January 2012	January 2012
			October 2012 to February 2013 inclusive	October 2012 to January 2013 inclusive
2011-mid 2018Reference = 2011 + 2012	Scotland—fallen stock (Figures 11A,C,E, respectively)	April 2013	April 2013	
		April and May 2015		
		April and May 2016		
				September 2016 to January 2017
			April 2017	
				August, September, and November 2017
		April 2018	April 2018	February to April 2018
	England and Wales combined—fallen stock (Figures 11B,D,F, respectively)	April 2013	April 2013	January to April 2013 inclusive
			August 2015	
				March 2016
				October and November 2016
				November 2017
				March 2018

There were variations by postcode area in the overall number of collections, SSUs, use of bulk collections and thus AUs, with higher numbers predominantly in the central and southern areas of Scotland that encompass higher sheep and sheep-holding densities. Annual trends also varied by postcode area (data not shown).

**Figure 6:**
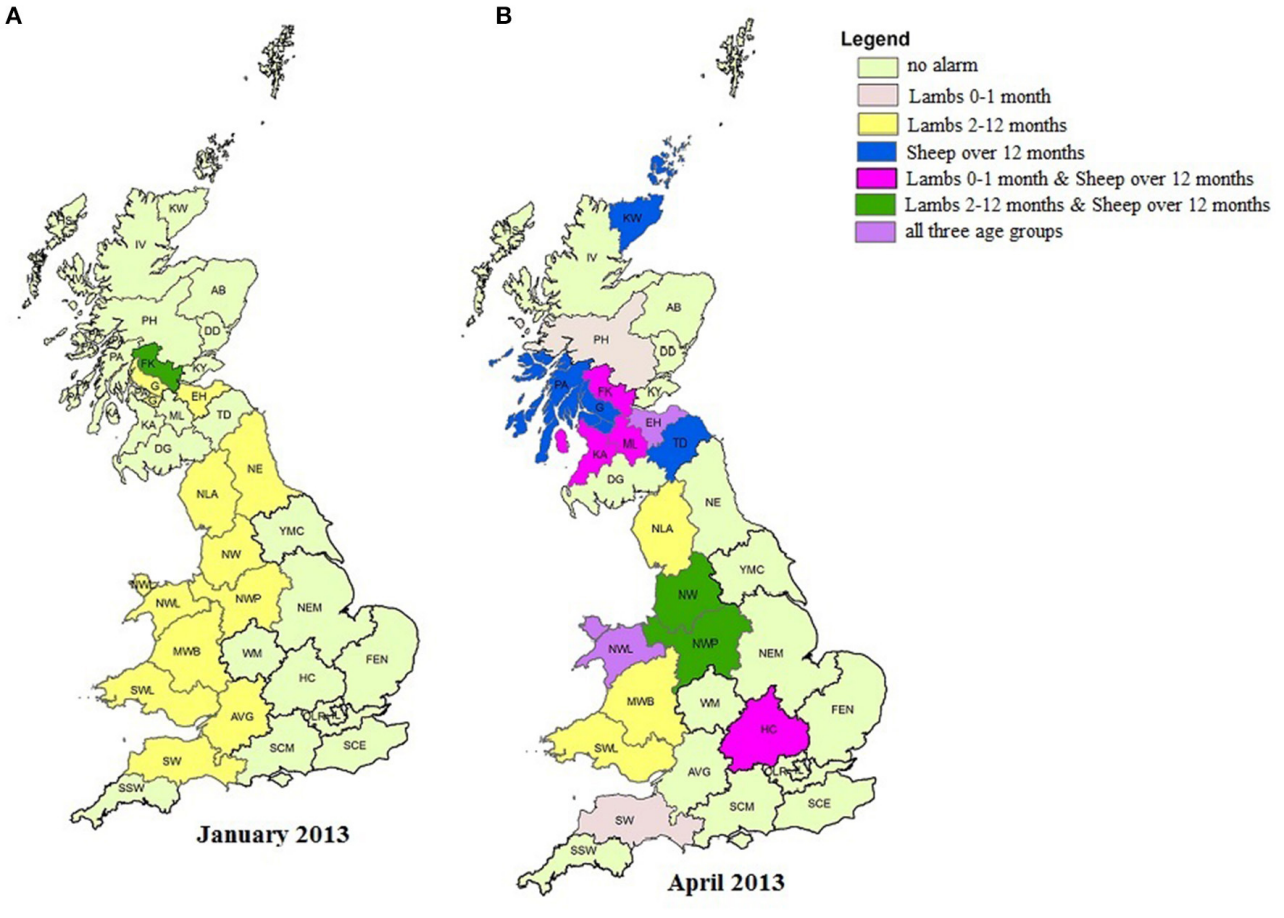
**(A,B)** Postcode area (Scotland) and regional area (England and Wales) alarms raised by the temporal aberration detection algorithm (TADA), by age group for the 2011–2014 fallen stock datasets for two calendar months: January 2013 **(A)** and April 2013 **(B)**. Ordnance Survey data © Crown copyright and database right 2017; Royal Mail data © Royal Mail copyright and database right 2017 and National Statistics data © Crown copyright and database right 2017.

At a postcode area level, the April 2013 alarm was apparent in five postcode areas for Lambs 0–1 month, of these four also had alarms in April 2013 for Sheep over 12 months. Four additional postcode areas had April 2013 alarms for Sheep over 12 months (Figure 6B). Additional alarms were triggered in other months, in other postcode areas for both age groups, while a number of alarms were triggered, in a variety of postcode areas for the Lambs 2–12 month age group (e.g., Figure 6A for January 2013—other data not shown).

*Validation of assumptions for conversion of fallen stock data to animal units.* Both working groups agreed that bags of lambs would be most likely to be used at peak lambing periods for peri- and neonatal deaths of 0–1 month lambs; that four would be considered a minimum for lambs in a bag and that 5 kg was possibly a little on the low side for the lamb weight. They also suggested that dead ewes weighed less than one would anticipate. No better method of allocation and classification was proposed.

##### Stage 2—England and Wales 2011–2014

As for Stage 1, subset analyses were completed to confirm that the multiple units needed to be included to provide an overall picture. This was the case in all analyses. The pattern of use of the multiple units varied between the two countries and years—using the full combination of “all animal” unit variables, for each of the three age groups, produced results that were comparable.

As in Stage 1, in all analyses, for all three age groups seasonal patterns with an annual cycle were seen.

*England (data not shown)*. The seasonal pattern and seasonal effects were again similar for English Lambs 0–1 month and Sheep over 12 months but differed for Lambs 2–12 months. The former had a peak between February and May with between 4 and 4.5 times the expected monthly average mortality in March and April for Lambs 0–1 month and ~2.5 times that for Sheep over 12 months. The February-May peak was more sharply defined for the Lambs 0–1 month than the Sheep over 12 months age group. The seasonal patterns and effects for Lambs 2–12 months were similar to those for this age group in Scotland being at their lowest in May and highest in the late autumn/winter months October to January. However, the English data did not show a plateau in the autumn period as seen in the Scotland data, while in England there was a monthly average mortality above 1 in September.

The average annual trend was similar for all three age groups although the magnitudes of the peaks and troughs differed slightly between them. They differed from the average annual trends in Scotland, being consistently upwards overall from 2011 to a peak in 2013, with 2014 being similar to 2011.

At a country level, the predicted threshold was exceeded and some alarms triggered in different age groups at a number of time points (Table 4).

*Wales (data not shown)*. The seasonal pattern and seasonal effects were similar to those seen in the English data for all three age groups. As per the average annual trend in the Scotland and England data, these rose to a peak in 2013 for all three age groups. However, while for Sheep over 12 months and Lambs 2–12 months overall 2014 was similar to 2011, for Lambs 0–1 month there was a decline, similar to the pattern observed in Scotland, but different from the rise observed in England. Again a number of alarms were triggered in different age groups (Table 4).

*England and Wales combined*. The seasonal patterns, seasonal effects and annual trends (Figures 2, 3, 4B,D,F) for each age group were subtly different from those in the Scottish dataset (Figures 2, 3, 4A,C,E).

By combining the England and the Wales datasets, it became possible to look at spatio-temporal patterns on a regional area basis. Over the study period, the majority of the estimated Lambs 0–1 month AUs are from two regional areas (HC and NLA) with the majority of Sheep over 12 months AUs coming from sheep-dense regional areas (MWB, NLA, NWL, and SW). The majority of the estimated Lambs 2–12 month AUs are also from four of these areas (NLA, MWB, and SW with NWL being replaced by NW).

The average annual trends vary between regional areas and age groups (data not shown). For Lambs 0–1 month, in several regional areas the estimated number of AUs collected increases on an annual basis, with an additional peak in 2013. This is particularly apparent in four areas. In some regional areas there is a fall in 2014, while in one the number drops from the reference year of 2011 and is much lower in all of the next 3 years. For Sheep over 12 months, on an annual basis, the number collected from most of the regional areas is fairly steady, although several regional areas saw an increase in numbers in 2013. In two regional areas, the estimated number of AUs collected increased on an annual basis, while in one, apart from 2013, there is a decline over the time period. For Lambs 2–12 months, the estimated numbers of AUs collected are again generally fairly steady, or gradually increase, sometimes with indications of peaks in 2013. However, in one regional area, the number collected decreased from 2011; in two there were increased numbers collected in both 2012 and 2013, while in another one there was a steady year on year increase.

A number of alarms were raised in various regional areas and age groups over the time period (see Figures 6A,B, for examples). Some contribute to the overall England and Wales alarms e.g., the Lambs 0–1 month September 2013 overall alarm appears to arise from a single regional level alarm that month; the October 2012 Sheep over 12 month overall alarm is reflected by regional alarms in this age group and month in three areas. It is also possible to identify areas where alarms were raised in a number of age groups, either coincidently, or in subsequent months e.g., The alarm for Lambs 2–12 months, seen in the SW in November 2013, follows alarms in the same area in September and October 2013 for Lambs 0–1 month and Sheep over 12 months, respectively (data not shown).

##### Recommendations from Stages 1 and 2

Twenty-one recommendations arose from Stages 1 and 2 (Table SM4). These included suggestions for improvement of the data, other actions required to facilitate use for surveillance purposes and for further investigations were made to the data-providers.

#### SRUC VS Fasciolosis Data 2011–2014

##### SRUC VS fasciolosis data- descriptive analysis

There were 1,036 eligible ovine diagnostic submission records in the 4 year study time period i.e., cases of either VIDA372 (acute) or VIDA373 (chronic). Almost three-quarters had a primary diagnosis of chronic fasciolosis, while just under one in five (17%) had a primary diagnosis of acute fasciolosis. Some submissions were not of Scottish origin (Table SM5).

The 178 Scottish primary acute fasciolosis cases represented an estimated 249 AUs. Just over half of these were from sheep, or groups of sheep, that could be categorized as over 12 months of age (*n* = 98, 55%), over a third were from lambs between 2 and 12 months of age (*n* = 67, 38%) while the rest, less than one in 10, could not be categorized into one of these two age groups, given the available information (*n* = 13, 7%).

Approximately a third of these acute cases, at both submission and AU level, came from Dumfries and Galloway (DG) postcode area. Four out of five came from the five southern postcode areas (DG, TD, KA, ML, EH) that are high density areas in terms of the number of breeding ewes. Within each of these five areas, the number of cases (as AUs) was greatest in 2012.

The 702 Scottish primary chronic fasciolosis cases represented an estimated 2,150 AUs. Just over half of these diagnoses were from sheep, or groups of sheep, that could be categorized as over 12 months of age (*n* = 373, 53%), while just over a tenth were from lambs between 2 and 12 months of age (*n* = 74, 11%). The rest—over a third—could not be categorized into one of these two age groups, given the available information (*n* = 254, 36%). As for acute fasciolosis, just under a third of the chronic fasciolosis diagnoses came from the DG postcode area, although elsewhere a more even distribution across postcode areas was seen than with the acute fasciolosis diagnoses.

Unlike the acute fasciolosis cases, where there was a peak in 2012 in many postcode areas, the pattern of numbers per year, or annual effects, for chronic fasciolosis case varied widely, by postcode areas. These data were excluded from the time-series analysis due to the more indirect link with mortality in sheep than with acute fasciolosis.

##### SRUC VS fasciolosis data—time-series analysis

The majority of acute fasciolosis cases occurred between October 2012 and January 2013. At an animal level (AUs) there is a similar pattern to that of the submission-level cases, although there is an apparent additional peak in the former during the autumn of 2013 (data not shown).

**Figure 7:**
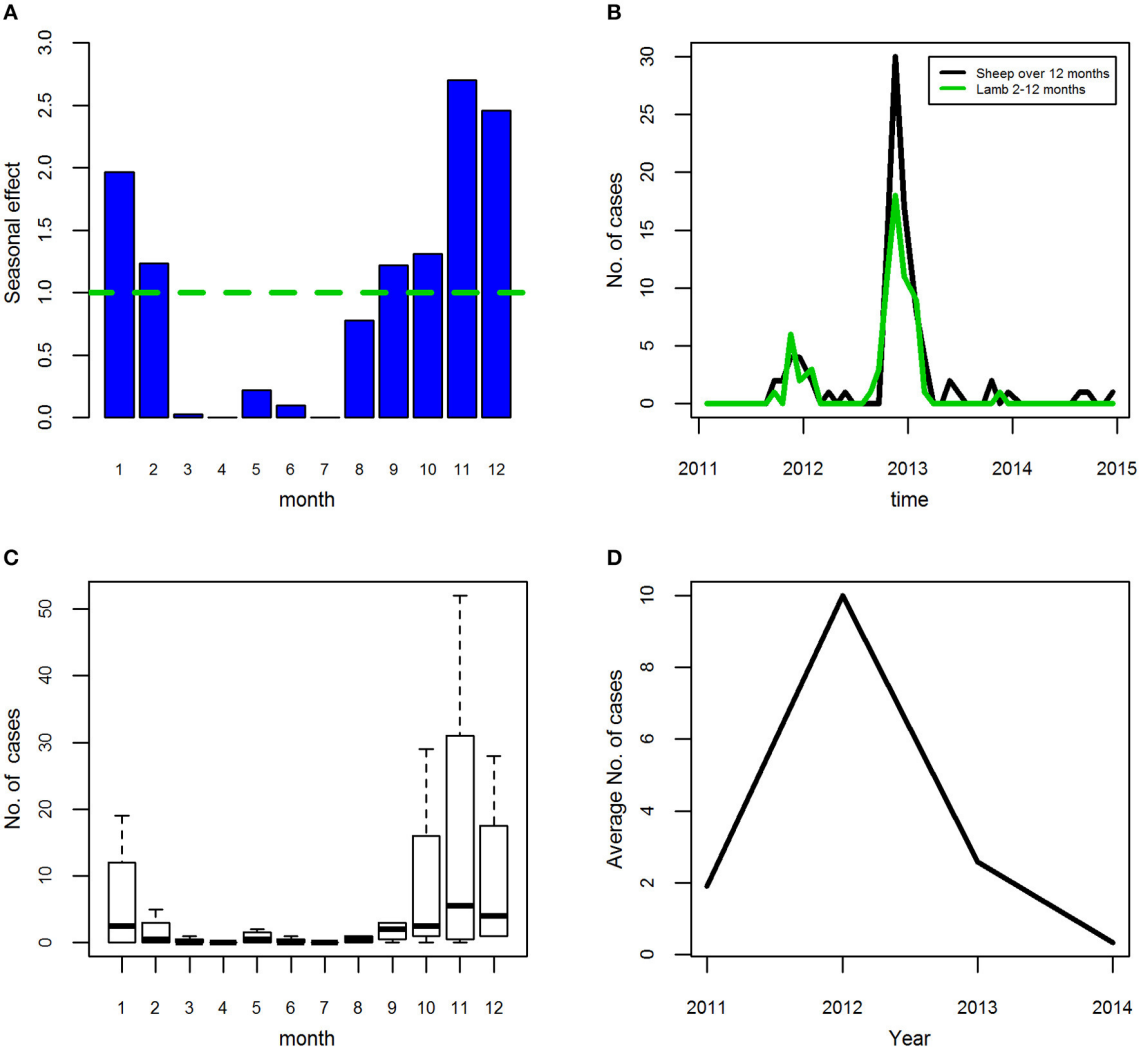
**(A–D)** Seasonal effects **(A)**, time-series **(B)**, seasonal pattern **(C)**, and annual trend **(D)** for ovine acute fasciolosis (VIDA372) diagnoses assigned at a submission-level (i.e., cases) in Scotland, 2011–2014 inclusive.

The seasonal effect for acute fasciolosis cases is <0.5 for the months of March to July with a rise starting in August (Figure 7A). This rise continues with increased seasonal effects being seen for each consecutive month until November, then declining again. The effect is similar at an AU level except that the October seasonal effect is larger than for cases.

Over the study period, the number of acute fasciolosis cases for Lambs 2–12 months was broadly similar to, but did not entirely mirror, that of the Sheep over 12 months of age group (Figure 7B). There are indications, for both age groups, of a seasonal pattern with an annual cycle that varies from year to year. The autumn 2013 peak in Sheep over 12 months is higher when AU level units, rather than submission-level cases, are considered.

Overall, using cases, the seasonal pattern has a peak between October and January (Figure 7C). At an AU-level, by age group the peak is similar but with subtle differences between the age categories. The average annual trend rose sharply from 2011 to 2012, and then dropped to lower than 2011 levels in 2014 overall (Figure 7D) and for both age groups (data not shown).

**Figure 8:**
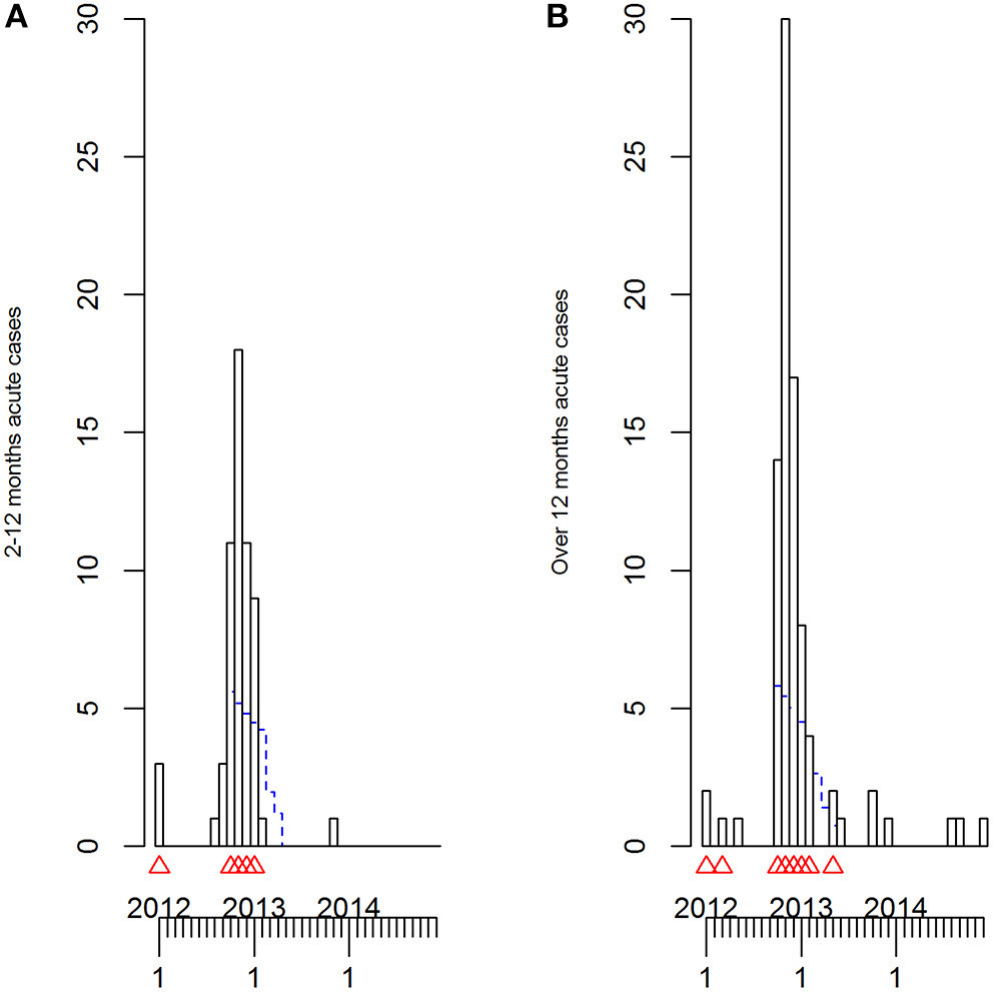
**(A,B)** The time-series plots and temporal aberration detection (TADA) for all ovine acute fasciolosis (VIDA 372) diagnoses assigned at a submission level (i.e., case) in Scotland, 2011–2014 inclusive for the two age groups: lambs 2–12 months **(A)** and sheep over 12 months **(B)**.

The TADA were only run at a country level due to the low number of data points. TADA alarms were raised at the same time points for both AUs and submission-level cases, using all primary VIDA372 records. These were in: January and March 2012; months between October 2012 to January 2013 inclusive and in May 2013. The TADA alarms at country level for Sheep over 12 months were the same as those for the all age analysis when using AUs, except for an extension of a month into February 2013 (Table 4). While with submission-level cases, for Sheep over 12 months, the March 2012, May 2013 and February 2013 alarms did not get triggered (Figure 8B). However, for the Lambs 2–12 months group, while similar to the all records analysis, the March 2012 and May 2013 alarms did not occur with either unit of analysis (Figure 8A and Table 4).

##### Recommendations from Stage 3

Six recommendations arose from Stage 3 (Table SM4). These included suggestions for improvement of the data and other actions required to facilitate use for surveillance purposes and for further investigation.

#### Fallen Stock Animal Units (AUs)

##### Stage 4−2011- mid-2018

As in Stages 1 and 2 subset analyses were completed to confirm that the multiple units needed to be included to provide an overall picture. This was the case, so the outputs from the categories including all the multiple units are presented here, as in previous sections.

**Figure 9:**
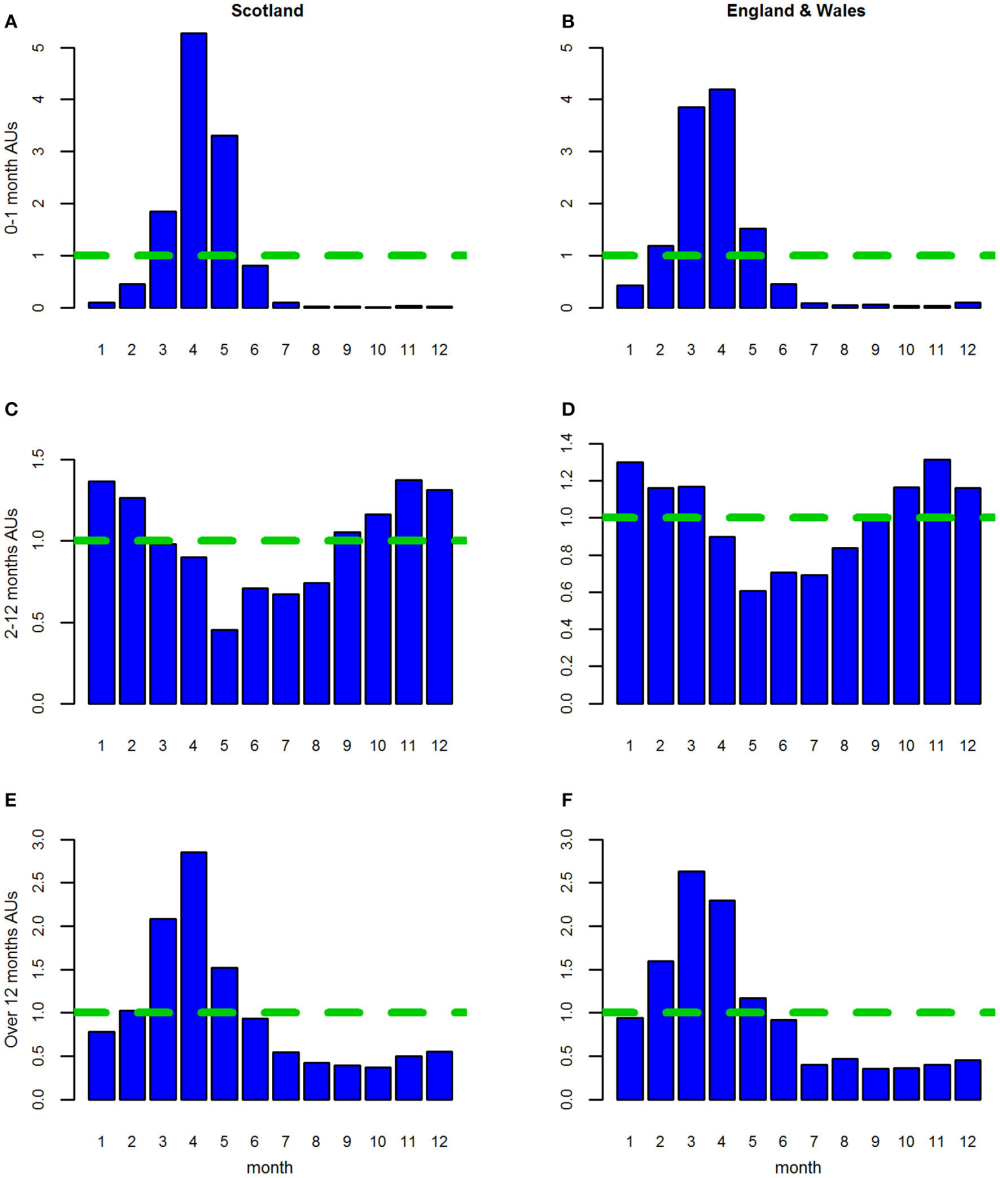
**(A–F)** The seasonal effects for the three age groups of estimated animal units (AUs) by country using the 2011 to mid-2018 combined fallen stock datasets.

*Scotland*. As in the shorter dataset, for all three age groups, in AUs, seasonal patterns with an annual cycle were seen. These were broadly similar for Lambs 0–1 month and Sheep over 12 months but differed for Lambs 2–12 months. The seasonal patterns (Figures SM1A,SM1C,SM1E) and effects (Figures 9A,C,E) varied slightly from those seen in the Stage 1, although the period where the seasonal effect is above 1 remained the same for each of the three age categories (Figures 9A,C,E). For Lambs 0–1 month, the peak was still in April (5x); however the effects were higher in May (3x) and March than previously (Figure 9A cf. Figure 3A). The peak for Sheep over 12 months was also still in April (~2.8x) and was higher in March than in Stage 1 (Figure 9C cf. Figure 3C). For both these age groups the seasonal effect was now lowest in October. The seasonal patterns and effects for Lambs 2–12 months were still lowest in May and highest in the late autumn/winter period although there are subtle differences in individual months between the two time periods (Figure 9C cf. Figure 3C).

**Figure 10:**
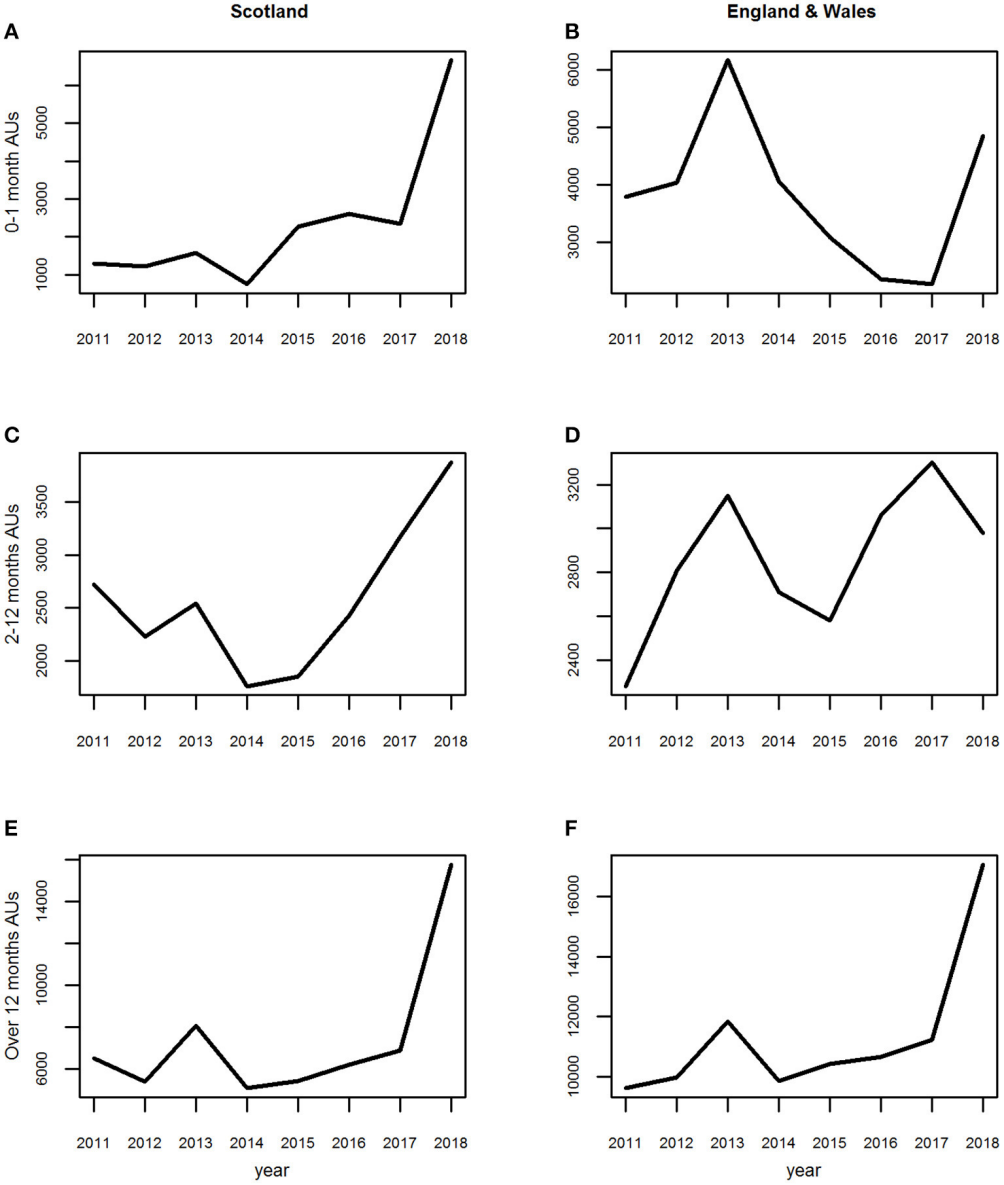
**(A–F)** The average annual trend for the three age groups of estimated animal units (AUs) by country using the 2011 to mid-2018 combined fallen stock datasets.

As previously, the average annual trend over the extended time period differed between the three age groups (Figures 10A,C,E). For Lambs 0–1 month following the 2013 peak and 2014 decline the numbers rose substantially above 2013 levels in 2015 to 2017, with a further dramatic rise in 2018 (Figure 10A). For Sheep over 12 months there was a low gradual increase from 2014 to 2017, with a subsequent dramatic rise in 2018 (Figure 10C); whereas for the Lambs 2–12 months, there was a consistent rise in trend year on year from 2015 to 2018, inclusive (Figure 10E).

**Figure 11:**
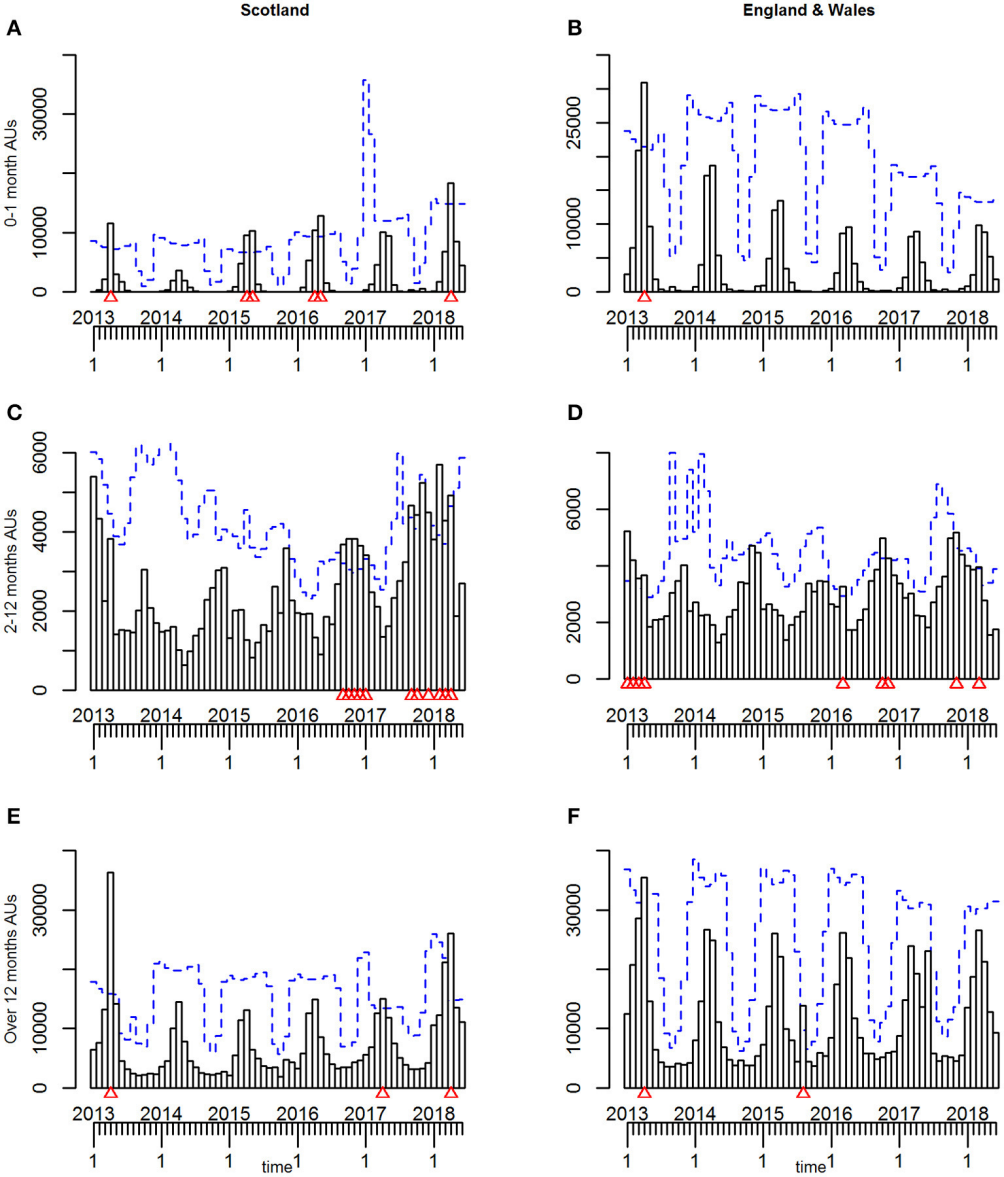
**(A–F)** The time-series plots and temporal aberration detection (TADM) for the three age groups of estimated animal units (AUs) by country, using the 2011 to mid-2018 fallen stock datasets, with 2011–2012 inclusive as the reference period.

At a country level, alarms were triggered in April 2013 for both Lambs over 0–1 month and Sheep over 12 months. There were additional statistical alarms triggered in the spring of 2015, 2016, and 2018 for Lambs 0–1 month and in 2017 and 2018 for Sheep over 12 months, the alarms (Figures 11A,E and Table 4). Alarms were triggered for Lambs 2–12 months in the autumn and end of the year 2016, again in some months of the same period in 2017 and the first third of 2018 (Figure 11C and Table 4).

As with the earlier dataset, there were variations by postcode area in the overall number of AUs and annual trends (data not shown). The most notable of these being an absence of any AUs from one area in 2018 and a 20x increase in the annual numbers of Lambs 0–1 month from one postcode area in 2015–2018 compared to the period 2011–2014. A corresponding increase in the magnitude of Sheep over 12 months from this area during 2015–2018 was not seen.

At a postcode area level with the new reference period, the April 2013 alarm was now apparent in six postcode areas for Lambs 0–1 month and 11 for Sheep over 12 months. Additional alarms were triggered in other months, in other postcode areas for both age groups. The spring 2015 and 2016 Lamb 0–1 month alarms were not confined to the postcode area with the increase in numbers noted above but were also raised in five and two other postcode areas, respectively. Nine areas had alarms for Lambs 0–1 month in April or May 2018, and eight for Sheep over 12 months in March or April 2018. Again, a number of alarms were triggered, in a variety of postcode areas for the Lambs 2–12 month age group (data not shown).

*England and Wales combined*. As per the shorter time period in Stage 1, the seasonal pattern (Figures SM1B,SM1D,SM1F) and effects (Figures 9B,D,F) were broadly similar for Lambs 0–1 month and Sheep over 12 months but differed for Lambs 2–12 months. The seasonal effects for the first two age groups remained similar to those seen before (Figures 3B,F c.f. Figures 9B,F), whereas both the seasonal patterns and effects for Lambs 2–12 months have changed slightly. While the effects are still lowest in May, they are now above 1 for October through to March, inclusive rather than September to February and the November peak effect, seen earlier, has been moderated (Figure 9D c.f. Figure 3D).

The average annual trend over the extended time period differed between the three age groups to an even greater degree than that observed in the Scottish data. For Sheep over 12 months the trend is similar to that seen in this age group in the Scottish data (Figures 10E,F), whereas, Lambs 0–1 month differ (Figure 10B c.f. Figure 10A). The average annual trend for Lambs 2–12 months in the combined England and Wales dataset also differs from that seen in this age group in Scotland (Figure 10D c.f. Figure 10C).

For the combined English and Welsh dataset, overall, only the April 2013 alarm remains for Lambs 0–1 month and Sheep over 12 months, while there is an additional alarm in August 2015 for the latter (Figures 11B,F and Table 4). For the Lambs 2–12 months there are still alarms in the first third of 2013, with additional alarms at a number of spring and late autumn periods in a number of years. Some of these are coincident with alarms in Scottish Lambs 2–12 months, others differ (Figures 11C,D and Table 4).

When looking at the annual numbers by regional area, the most notable variations are a substantial reduction in the numbers of Lambs 0–1 month per annum from one area from 2016 on and double the usual numbers of Sheep over 12 months from another area in both 2015 and 2017 (data not shown).

A number of alarms were raised in various regional areas and age groups over the time period. Some contribute to the overall England and Wales alarms and some don't e.g., seven areas have a Spring 2013 alarm for the Lambs 0–1 month, either just before, just after, or in April, while six areas have an alarm in this age group in March or April of 2018. For Sheep over 12 months, nine areas have a Spring 2013 alarm, there are scattered alarms in this age group in the late months of 2017 and early months of 2018 and only two regional areas with an August 2015 alarm, while there are scattered alarms elsewhere in neighboring months. In the Lambs 2–12 months, seven regional areas have alarms raised in either February or March 2018, while all but two regions have alarms raised in one or two of the months between January 2013 and April 2013 inclusive.

## Discussion

### Key Findings

In this study, an existing data source—business operational data from a voluntary fallen stock collection service—was investigated, to determine if these data could be used as a proxy for the mortality experienced by the British sheep population, in the absence of any other appropriate data source for this species. Despite various limitations (discussed below), these data could, with appropriate domain expertise, be converted into a useable format. They did reflect the seasonal pattern expected from knowledge of the British sheep production-year calendar (47); the spatial distribution of the sheep population of GB (48, 49) and the slight variations across Britain associated with the different sheep management systems and its stratified nature (50). Statistical aberrations were detected in relevant areas and age groups at the time of a known extreme weather event that occurred during March and April 2013 at peak lambing time (51). Similarly, the effects of a more widespread weather event slightly earlier in the spring of 2018 were detected (52). In addition a number of statistical alarms were raised in regions at specific time-points early in the study period that potentially reflected other known challenges. This prompted the investigation of the SRUC Veterinary Services fasciolosis diagnostic data. The primary aim here was to determine if it could be utilized in parallel i.e., to see if it precedes, coincides or assists in the explanation of specific mortality alarms that have been detected and thus facilitate interpretation; albeit based on an *a priori* hypothesis that fasciolosis contributed to the mortality. These data brought their own limitations (also discussed below), one of which is the sparsity of the data, although they do reflect both the annual life cycle of liver fluke and the sensitivity of this parasite to changing weather conditions from year to year (53).

Based on the limitations identified, a number of recommendations were made for each data source. These recommendations were framed in the context of development of these data to facilitate their use for animal health surveillance purposes, either as a surveillance system component (38) or as contextual background i.e., “intelligence” (23, 27). Most would require improvements to data collection and provision. While the re-analysis in Stage 4 addressed the need for a longer temporal period [Recommendation (R) 13, Table SM4], the updated dataset also provided evidence of changes that addressed two other recommendations; changes which facilitate the interpretation of any statistical alarms (R4, Table SM4) and which provide potential for future analysis at a more relevant time-scale (R3, Table SM4).

### Challenges; Limitations and Interpretation

For both data sources, one of the primary challenges was the lack of suitable denominator data. While the initial fallen stock membership files provided some insights into aspects of the data that would assist interpretation of patterns, trends and alarms, they were not appropriate for use as denominator data. This was due to the lack of information on the livestock species, or numbers, at a Collection Point and the “snapshot” nature of the data. The unique identifier for a member was specific to this system, therefore collection records could not be linked to other data sources such as the June Agricultural Census or December Sheep and Goat Inventory to acquire additional information. Using the total number of sheep holdings [as defined by County/Parish/Holding (CPH) numbers] in either of these data sources would not be appropriate due to a number of factors. These include: the voluntary nature of membership—not all farms/holdings with sheep will be members; the availability of alternative collection services—not all collectors will be members; variability in the use (if and when available) of these alternative collection services; associated drivers for changes in membership and use of NFSCo collectors, plus the existence of and variability in uptake of the derogations for remote areas (40). The latter particularly applies to the Scottish Highlands and Islands (41). This is evident in the data and means that significant contributions from these areas might be missed and events go undetected. It is essential that the potential for changes in any, or all, of these factors need to be taken into account first when interpreting outputs.

In the VS fasciolosis dataset, CPH numbers were available for the majority, but not all, of the diagnostic submission records. While CPH is not a perfect identifier and new systems are in development (54), it is (probably) the best that currently exists. However, it is known that not all holdings would submit to the VS network (3). Thus, in addition to the requirement to analyse the VS data in a comparable manner to the fallen stock data, again neither of the two statutory demographic data sources would be a suitable denominator.

For the fallen stock data, the second challenge was the conversion of the relatively standardized recording unit used for business purposes (SSUs) into something that more appropriately measured the mortality experienced, either at a member, flock, collection point, or animal level. The former three were not feasible, given the aggregation of the data and lack of denominator information. Simple multiplication was used to change the 10+ animals of a stated age group into what is a recognized under-estimate; the other multiple units; bags, kg, liters, skips presented more of a problem. The solution was to plot frequencies and assess the impact of inclusion, exclusion on the analyses, in conjunction with discussion with those who have indirect and direct experience of using such services. Exclusion of liters and unweighed skips due to the infrequency of their use, plus exclusion of Dolav® sheep per kilo based on their specific but relatively consistent use, was strengthened by the additional likelihood that the time of collection for these SSUs did not necessarily correlate as closely with the time of death of the animals that are within the collection. It would, however, be advisable to monitor usage of such bulk SSUs. If these changed in frequency substantially, this in itself could raise an alarm to be investigated (R14, Table SM4). Reasons for use may vary from needing a larger container due to a true increase in mortality to specific preferences for storage of fallen stock by members, or provision of services by collectors. Either way their increased use could result in corresponding reductions in use of other SSUs, or vice versa, their decreased use might result in increased usage of other SSUs, with the potential for production of false-positive alarms. One such change was observed in one of the Scottish postcode areas, although it involved the Sheep/lambs per 10 kg SSU. From 2015 on there was a huge increase in the number of Lambs 0–1 month SSUs. There was a coincident decrease to almost 0 in the use of the Sheep/lambs per 10 kg SSU. Alarms were raised in this postcode area for the Lamb 0–1 month age group during spring 2015 and 2016; however, they were not confined solely to this area.

The frequency distribution of the 10+ bags of lambs over time was very similar in shape to that of the single lambs 0–1 month age group, in the Stages 1, 2, and 4 datasets. SAC Consulting Farm Business Services colleagues confirmed that this was the age group for which bags would be used and provided the initial estimate of a minimum of four lambs per bag. This was validated as a likely under-estimate by two other industry groups. As all of these groups were Scottish based, it would be worthwhile ensuring that this assumption holds for England and Wales (R10, Table SM4). The addition of the AUs resulting from the 10+ Lambs 0–1 month and the 10+ bags of lambs did not alter the general shape of the time-series, trends and patterns for this age-group but did alter the magnitude of effects. It was, therefore, necessary to ensure that these multiples were included, while accepting that the assumptions used still lead to under-estimation of AUs in this age group and that it is also reliant on the assumption that bag-use behavior is consistent over time. This underestimate might lead to a potential for a reduction in the ability to detect an aberration when one exists and could be improved by additional recording of the actual number collected, rather than just “10+” (R7, Table SM4). More difficult was the need to incorporate the relatively frequent but very area-specific use of 10 kg of Sheep/lamb SSUs. Whilst being relatively confident from the frequency distributions over time that these are more likely not to be 2–12 months old lambs, the assumptions made based on total weight frequencies are open to challenge. The potential for variable weights of lambs and sheep over 12 month, presumed to be ewe carcases, due to both breed variations and decomposition on storage is acknowledged. This is of particular importance with regard to the country-level alarm raised in the Stage 2 analysis of the Lambs 0–1 month age group in both England and Wales for September 2013. This would not be a time of year when typically a large number of 0–1 month old lambs would be expected to be on the ground, even when this category will include stillborn and unborn i.e., aborted fetuses. Potential misclassification includes lambs of the 2–12 months age group being bagged and adult sheep contributing to kg collections. No emerging threats were noted at this time (55).

The European standard approximate average bodyweight used for adult sheep in estimating antimicrobial consumption (ESVAC) is 75 kg (56). The lower value chosen of 50 kg may be more appropriate for carcases, or a reflection of the Scottish focus of the Stage 1 analysis i.e., more hill and upland sheep, rather than lowland sheep. Again, validation of this assumption for England and Wales (R10, Table SM4) may be worthwhile. Sensitivity analyses with different weight distribution algorithms are also a possible approach. However, given the approximate nature of the estimates already being made with imperfect data, that may be more of an academic exercise than a beneficial use of resources. Sensible interpretation would possibly maximize reliability (57).

A number of aberration detection algorithms—Farrington (44), improved Farrington (58), Binomial CUSUM (59), Negative Binomial (60), CUSUM (61, 62) and BODA (63)—were considered. The use of count data with an initially short study time period drove the choice of the original Farrington method (44). Seasonality is taken into account by using a subset of the available data as a reference, with the added assumption that there is no aberration in the reference period. An aberration (or statistical alarm) is detected at a single time point, if the count exceeds a threshold value, however, the method does not detect sustained shifts as it does not accumulate evidence over several time points. Additionally, only a window of historical values is taken for estimation of the threshold values, with no values taken from the current year. Despite these shortcomings, it is widely used in human public health surveillance (44, 64) as it is easy to implement and tackles major issues encountered with surveillance data; adjusting for over-dispersion of data, past outbreaks, trend and seasonality.

The choice of reference year can affect the outcome, especially when the assumption that there is no aberration in the reference period may be difficult to achieve in practice. There was little potential for choice other than 2011 in the short 4 year time period of the Stages 1–3 analyses. There was concern that this might not be a “typical” year, especially for Scotland, given the decline in average annual trend between then and 2012, although this may possibly have been driven more by membership changes than mortality effects. Despite this and the Schmallenberg incursion in the South-East of England in 2012 (55), the alarms seen in Lambs 0–1 month and Sheep over 12 months, in Scotland and England and Wales for April 2013 were observed in both the shorter and longer temporal period analyses, as were the alarms in the first quarter of 2013 for the 2–12 months lambs in England and Wales, suggesting a degree of stability.

The initial aggregation of the fallen stock data at a monthly level was sufficient to explore the utility of the data. It may also be sufficient for the provision of context, i.e., situational awareness and to evidence anecdotal observations, or to answer policy questions (22, 23). There is always going to be an initial delay between fatalities and collection and then collation, provision and analysis of data, as observed in cattle data (32, 65). This is likely to be longer in a non-statutory system but may be improved with the introduction and increased usage of digital technologies. The finer timescale of the Stage 4 data (R3, Table SM4) aids interpretation of observed alarms at the monthly aggregation-level and raises the possibility of further analyses. The most appropriate time-scale to use needs to be investigated to optimize the noise-signal ratio. The mortality experienced in a population would fit the definition of syndromic surveillance -“Surveillance that uses health-related information (clinical signs or other data) that might precede or substitute for formal diagnosis” (37, 38) if a semi-real time system could be implemented. For many disease conditions it would be hoped that existing surveillance systems would pick up incidents earlier. However, new and re-emerging threats might come from a variety of sources. These include factors such as: management changes; economic factors; food and forage prices, availability and nutritional content; extreme weather events; climate change; changes in welfare etc. These may come in a variety of different forms; be they insidious, sporadic, repetitive and intermittent, or continuous. Death is one of the few certainties of life, although the cause may vary. Mortality within a population may be affected by many of these potential threats, therefore, an increase in mortality may be an indicator of reduced health of a stable population and vice versa. It is also possible to visualize conceptually and theoretically how different data sources may be brought together for syndromic surveillance, using “syndromes” made up of multiple components, of which mortality could be one, particularly in a species such as sheep that have a propensity to be “found dead” (43). Of course, to extrapolate the fallen stock mortality experience from NFSCo members to the national sheep flock would require the assumption that the mortality experience for non-members is the same as that for members. This would be a dangerous assumption to make without further investigation and understanding of the drivers behind membership and a better understanding of the attributes of members' flocks, stock numbers and type at collection points. Integration with data from other fallen stock companies could, potentially, provide increased coverage and improve representativeness of the mortality experience of British livestock populations. This would require either unique identifiers, or sufficient, standardized identifiers in each data source to facilitate the linkages. It would also require a degree of standardization of the collection process and data recording of the animal units collected.

While many potential threats will not respect man-made boundaries, given the lack of point location data and concerns about confidentiality, plus the variation in the distribution density of livestock holdings and the British sheep population (48, 49), some degree of spatial aggregation and regionalization is required. The choice then becomes what scale is appropriate and what boundaries to use. Here this was driven by the recording and structure of the data provided. The Scottish Government's Agricultural regions (66) are broadly similar to the postcode areas. By using the latter no further data manipulation was required. Due to the much larger numbers and border peculiarities of the English and Welsh postcode areas, application at this level was not possible and new regional areas based on multiple postcode areas had to be generated. The advantage is that they were formed using domain expertise; taking into consideration the density distribution of the sheep population and holdings, likely differences in management systems, urban and suburban areas, natural barriers and east-west/north-south weather influences, so as to have some meaning in this context. The disadvantage is that they could be considered to be arbitrary and subjective. Nomenclature of Territorial Units for Statistics (NUTS) codes (67) at level 1 provide only 10 areas, with no distinction between North and South Wales, whereas the NUTS 2 areas would be too fine (35 areas). Additionally, further data processing would be needed and they may not have been possible to assign. Time-series analysis is required at both the larger country level and at the smaller regional level, run in parallel, to facilitate understanding of the underlying processes and aid interpretation of any alarms raised. If analyzed only at a country level, there is the danger of missing anomalies that affect specific areas with insufficient magnitude to trigger a countrywide alarm i.e., that would be “washed-out” in the larger picture. However, it is plausible that a country-wide alarm might be triggered by small regional increments that are not detected by the regional analysis but that add up to an incident overall. At a regional level the disadvantage is that the data is being split further and, especially in areas with low numbers of AUs, it may thus result in more false-positive alarms. As statistical alarms are only indicators, raising alerts of the need for further investigation, this then becomes a matter of resource availability and allocation and signal optimization (14).

Many of the limitations and challenges identified with the VS fasciolosis data mirror those in the fallen stock data, with the added issue of the paucity of data points. Some could be addressed through improved data quality and management. Others will require discussion and determination of what is most appropriate to use in order to answer the specific surveillance question that is postulated. The ones of most importance—for these types of time-series and aberration analyses are accurate capture and recording of the: number of animals included in a submission; number of animals in the submission that were allocated the specific diagnostic code; ages of animals (where that has a bearing on management, frequency, pathogenesis, or disease process and control strategies); first or repeat submission from an epidemiological unit and the flock/holding identifier for linkage to other data sources. Many of these are being addressed via the introduction of a new LIMS. However, as the primary purpose of the laboratory network is to identify and mitigate the effects of disease outbreaks, so recording of number of animal affected or examined is not of primary importance to reach a diagnosis at flock level. Therefore, the optimum units to use for the types of analysis investigated here will need to be agreed; whether it is animal unit or submission record, herd, flock, or holding level. This may need to be done at an individual diagnostic code, or syndrome level. For acute fasciolosis there was only a slight difference between estimated AUs and submission level analysis. This is likely to be due to the types of sample submission that lead to such a diagnosis i.e., found dead, or wasting submission of single animal carcase; rather than the submission of multiple feces samples for a chronic fasciolosis diagnosis. It was the timeliness of mortality associated with acute fasciolosis in sheep and the knowledge that 2012/13 had been a particularly bad year for livestock fasciolosis (55) that drove the choice of this as the exemplar condition for investigation. Alternative methods of analysis will need to be trialed to optimize the use of VS diagnostic data, in order to facilitate their use as surveillance intelligence. A first line, in parallel with mortality from fallen stock data, could be monitoring species-specific carcase submission levels for post-mortem, followed by cause-specific diagnostic code analyses when warranted. However, recent changes to the Disease Surveillance Center network (68, 69) may mean that existing data are no longer valid for reference. A better understanding of the drivers of the number of animal units per submission would be useful, although this may also be affected by the network changes.

### Advance and the Future

Despite the stated limitations, for the first time (in the authors' knowledge), population-level ovine mortality trends from fallen stock have been investigated. These studies have demonstrated that there is potential for imperfect data—voluntary fallen stock data collections—to contribute to surveillance intelligence provided the question is clearly defined and an informed approach is used for interpretation. The need to make better use of such data has been recognized for some time (4) and re-iterated recently (27). There is really no justification for the collection of data if it is not converted into information. However, when data are originally collected for a different purpose then not only the resources but a range of skills and subject-specific knowledge are required, working collaboratively with those involved in collecting and collating the original source data, to convert them into useful information (36). If it is to be of value and sustainable, operational implementation of any surveillance intelligence system will require cross disciplinary interactions and adequate resources; both for development and thereafter, as well as sufficient planning both of how to respond to statistical alarms and how decisions are made to deploy further investigative resources and/or apply mitigation measures (57). Although data and the technology to exploit them exist, human aspects will have to be factored in for meaningful progress to be made (70).

The fallen stock data could also be used to improve animal health surveillance in other ways. For example, if the delivery of collected fallen stock is known and if this is relatively stable i.e., collections from points A usually go to fallen stock center B; then it should be possible to inform and optimize the sampling design for targeted surveys using fallen stock material to estimate specified conditions (71, 72). Alternatively, the aggregated, population level, estimates of the frequency of occurrence of conditions identified emerging from farmer and/or veterinary practitioner requested, post-mortem (PM) of fallen stock at fallen stock centers (73) currently suffer from a large range of challenges and bias. To interpret this data meaningfully one needs to understand the relationships between the outputs, the data collection methods, the sampled population, the source population and national livestock populations. Analyses that aid understanding of the spatial distribution of fallen stock collection data could facilitate its use to guide improved interpretation of fallen stock acquired PM data (72). Furthermore, the recording of a standardized, categorized, member-derived “reason for death” at collection (R12, Table SM4) could only improve the interpretation of any observed changes in trends, patterns and any alarms with syndromic information. Whatever the direction of any future use of these existing data, these studies provide a foundation, or proof of concept; further development will be required before a functional system can be implemented. However, there is potential for use of these data as: a proxy measure for mortality in the sheep population; complementary components in a future surveillance system, and to inform the design of additional surveillance system components.

## Data Availability Statement

Data have been obtained from a third party. The data analyzed in this study were obtained from National Fallen Stock Company and from SRUC Veterinary Services. Requests to access these datasets should be directed to the corresponding author for forwarding to the appropriate contact.

## Author Contributions

ST conceived, designed, and led the studies, with contributions from JE (All stages), CC-G (Stages 1 and 2), and FB (Stage 3). FB contributed to collation and extraction of the SRUC Veterinary Services data (Stage 3). On receipt JE prepared all datasets and performed the statistical analysis. ST and JE contributed to the interpretation of all analyses, CC-G to interpretation of Stages 1 and 2, and FB to Stage 3. GG internally peer-reviewed the Stage 1 project report and helped secure Strategic Research Programme funding. With contributions from JE and CC-G, ST planned, drafted, and wrote the manuscript except for the statistical analysis sections, which were written by JE. CC-G, JE, and FB contributed to manuscript revision. All authors read and approved the submitted version.

### Conflict of Interest

The authors declare that the research was conducted in the absence of any commercial or financial relationships that could be construed as a potential conflict of interest.
