# Supplementary material for: Improving the Utility of Voluntary Ovine Fallen Stock Collection and Laboratory Diagnostic Submission Data for Animal Health Surveillance Purposes: A Development Cycle
Source: Front Vet Sci. 2020 Jan 24;6:487. doi: 10.3389/fvets.2019.00487 (PMC6993589; doi:10.3389/fvets.2019.00487)
Supplement: Supplementary file 1 [file Data_Sheet_1.docx]

Supplementary Material

# Supplementary Data

N/A

# Supplementary Figures (1) and Tables (5)

Table SM1: Potential combinations of sample type and submission reason that may be of relevance to liver fluke infections and associated implications for the diagnostic process and interpretation in terms of the disease process.

| Reason for submission | Sample type | Why assigned? | Diagnostic code applied | Stage of infection/disease process |
| --- | --- | --- | --- | --- |
| DIAG | PM  (post-mortem) | Tested at VIO’s discretion as part of investigation – a gross PM finding | If positive,  VIDA CODE 372  Acute fasciolosis  Based on:  Clinical history plus any of the following -   1. Gross pathology 2. histopathology | *migrating immature fluke*  Typical clinical history is “found dead”  Other presenting signs such as Wasted, Death, Diarrhoea may also be recorded |
| DIAG | PM  (post-mortem) | Liver fluke test/investigation requested | If positive,  VIDA CODE 373*  Chronic fasciolosis  Based on:  Clinical history plus any of the following -   1. Trematodes in the liver at PM examination 2. Liver fluke eggs in faeces 3. Positive coproantigen ELISA 4. histopathology | *liver fluke eggs = laying adults present*  Typical clinical history includes: Wasted, Death, Diarrhoea  Occasionally, though not truly valid for VIDA code – ‘healthy’ or ‘unknown’  * Possibly also diagnosed as an incidental finding where the cause of death remained unclear |

Table SM2: The 16 Scottish postcode area codes

| Postcode area code (PCA) | Name of PCA | Postcode area code (PCA) | Name of PCA |
| --- | --- | --- | --- |
| ZE | Shetland | FK | Falkirk |
| KW | Kirkwall | KY | Kirkcaldy |
| HS | Outer Hebrides | G | Glasgow |
| KW | Kirkwall | EH | Edinburgh |
| AB | Aberdeen | KA | Kilmarnock |
| PH | Perth | ML | Motherwell |
| DD | Dundee | TD | Galashiels |
| PA | Paisley | DG | Dumfries |

Table SM3: The English and Welsh postcode area codes and names and their aggregation into 18 larger regional spatial units, or regional areas for analyses.

| Area | Regional Name | Name of Postcode Areas included | | Postcode Area designation | |
| --- | --- | --- | --- | --- | --- |
| NE | North East | Cleveland  Darlington  Durham | Newcastle  Sunderland | TS  DL  DH | NE  SR |
| NLA | North & Lakes | Carlisle  Lancaster |  | CA  LA |  |
| YMC | York & Mid | Harrogate  Hull  Leeds | Wakefield  York | HG  HU  LS | WF  YO |
| NW | North West | Blackburn  Blackpool  Bolton  Bradford  Halifax  Huddersfield | Liverpool  Manchester  Oldham  Preston  Warrington  Wigan | BB  FY  BL  BD  HX  HD | L  M  OL  PR  WA  WN |
| NWP | North West & Peak | Chester  Crewe  Derby  Sheffield | Stockport  Stoke-on-Trent  Telford | CH  CW  DE  S | SK  ST  TF |
| NWL | North Wales | Llandudno |  | LL |  |
| NEM | North East Midlands | Doncaster  Leicester  Lincoln | Nottingham  Peterborough | DN  LE  LN | NG  PE |
| MWB | Mid Wales & Borders | Hereford  Llandrindod Wells  Shrewsbury |  | HR  LD  SY |  |
| WM | West Midlands | Birmingham  Coventry  Dudley | Walsall  Wolverhampton  Worcester | B  CV  DY | WS  WV  WR |
| FEN | Fens | Cambridge  Chelmsford  Colchester | Ipswich  Norwich | CB  CM  CO | IP  NR |
| HC | Home Counties | Hemel Hempstead  Luton  Milton Keynes  Northampton | Oxford  St Albans  Stevenage | HP  LU  MK  NN | OX  AL  SG |
| SWL | South Wales | Cardiff  Newport  Swansea |  | CF  NP  SA |  |
| AVG | Avon & Glos | Bath  Bristol  Gloucester | Swindon | BA  BS  GL | SN |
| OLR | Outer & London Ring | Bromley  Croydon  Dartford  Enfield  Harrow  Kingston-upon-Thames  Romford | Slough  Southall  Southern OS  Sutton  Twickenham  Watford | BR  CR  DA  EN  HA  KT  RM | SL  UB  SS  SM  TW  WD |
| SCE | South Coast East | Brighton  Canterbury  Redhill | Rochester  Tunbridge Wells | BN  CT  RH | ME  TN |
| SCM | South Coast Mid | Bournemouth  Guildford  Portsmouth | Reading  Salisbury  Southampton | BH  GU  PO | RG  SP  SO |
| SW | South West | Dorchester  Exeter  Taunton |  | DT  EX  TA |  |
| SSW | South South West | Plymouth  Torquay  Truro |  | PL  TQ  TR |  |

Table SM4: The 21 recommendations from the Stage 1 & 2 analyses of the fallen stock data

| Recommendation | Type | Action | Issue Addressed |
| --- | --- | --- | --- |
| Fallen stock datasets Stage 1 & 2 (Fill – changes seen in Stage 4 data-set | |  |  |
| 1 | REC/FIW | Further information about the NFSCo systems would enable an assessment of how technologically feasible this would be.. | It could be possible to set up a semi-real time, automated aberration detection system based on the detection of temporal aberrations in the count data. |
| 2 | REC | Sufficient resources would need to be made available to promptly investigate the factors and possible causes that result in the statistical alarm being triggered. This needs knowledge of the data, the collection process and the industry, as well as of potential causes such as weather and disease processes. | Generation from the data of additional information that is either not then effectively utilised, or is mis-interpreted; achievement of benefit/impact |
| 3 | REC/EDAP/FIW | Provision of the data on a finer time-scale and then further investigation will be required to determine exactly which time period is best; to balance the benefit with analytical and other issues that might arise. | To fulfil an effective early warning role for the detection of temporal aberrations, the analyses need to be run in a timely manner with the data aggregated appropriately. Monthly is too long, although this may be sufficient for situational awareness. |
| 4 | EDAP | Provision of Sheep Services file at a finer level i.e. each unique member-collection point at full postcode level | Improved interpretation of any observed changes |
| 5 | REC/FIW | Categorisation of England & Wales into new regional areas has been achieved. Validation that these spatial areas are appropriate is required from English & Welsh industry representatives. | Full postcode areas are not suitable for spatial and spatio-temporal analyses in England and Wales. This has been resolved by categorization into new regional areas. |
| 6 | REC | Methodology and algorithms to achieve conversion of the business data to estimates of the total number of animal units collected per month have been derived in the study. There are a number of possible refinements that could be made (PRecommendations (R) 7-11) | Conversion of the business data provided into estimates of the total number of animal units collected per month is required to more accurately reflect the mortality experience for that age group of sheep. |
| 7 | DIRADC | Record actual number of bags collected when over 10+ | Reduce underestimation of animal units collected |
| 8 | DIRADC/FIW | Record an indication of the age group of sheep in container/bag for lambs and in by-weight collections and proportion of each age-group when mixed) | Check the validity of the assumptions that have been made in converting these Species Service Unit (SSUs) to animal units |
| 9 | FIW | Investigate the variation in the number of lambs/per bag | Check the validity of the assumption that have been made in converting this SSU to animal units |
| 10 | FIW | Validation exercises are needed with appropriate industry representatives from England & Wales for the estimation from SSUs to animal units | Test whether the assumptions used in the analyses for Scotland hold for the England and Wales scenarios |
| 11 | DIRADC | Record reason for use of Sheep/lambs per 10LTR and Unweighed skip – Sheep, plus some indication of contents: numbers and age-group and the time of death (rather than collection) | Check the validity of their exclusion from current analyses |
| 12 | DIRADC | Record a crude member derived ‘reason for death’ at collection. | Improved interpretation of any observed changes.  Generation of baseline syndromic information. |
| 13 | REC/FIW | Repeat the analysis is a number of years | 2011 may not have been a ‘typical’ year: longer periods of available data are needed to improve the reference time period. |
| 14 | REC | The flagging of the use of specific bulk SSUs in new areas, or at new time points and the reasons therefore, could be considered as an alert that needs further investigation. | Improved interpretation of any observed changes |
| 15 | DIRADC | Provision of start and stop dates for members activity. | Inability to determine historically the number of ‘Active’ members at any time-point i.e. the temporal effects of membership retention and recruitment.  Assist in provision of a potential denominator. |
| 16 | DIRADC | Identification of each unique member-collection point at full postcode level; or provision of both bits of information in all datasets provided for analysis (i.e membership number and collection point full postcode). | Duplicated full postcode locations for multiple members; will improve interpretation of any alarms obtained at a higher aggregate level.  Assist in provision of a potential denominator. |
| 17 | DIRADC | Identification of species present at each unique member-collection point (CP) at full postcode level. | Improve/assist in provision of a potential denominator for individual species  This may be possible if ‘type of stock that is to be collected from this collection point’ is one of the pieces of information requested for membership registration. At a CP level would be ideal, or at a grosser level such as member would be an improvement. |
| 18 | DIRADC | Estimate of numbers of species present at each unique member-collection point at full postcode level; ideal, or at a grosser level such as member | Improve/ assist in provision of a potential denominator for individual species.  Not necessary if can link to other data sources that already contain this information: see unique identifier (CPH) issue below. |
| 19 | DIRADC | Identification of relationship between multiple collection points for a single member | Improve/assist in provision of a potential denominator |
| 20 | DIRADC | CPH of membership-collection point (i.e. property) | Improve potential denominator and link to other data sources. Other work has shown that – although there are issues with CPH – when combined with postcode and confidentiality issues are dealt with, this is the most useful, current way of achieving a link. Pseudo-anonymisation algorthims are now available to deal with confidentiality issues. |
| 21 | FIW | Investigation of the barriers and drivers for livestock producers to become members of NFSCo and recording of structural changes in the network | This would aid in interpretation of the data |
| 22 | REC/FIW | Determine if the points to which collected fallen stock are delivered are known and relatively stable, | It should then be possible to use these voluntary fallen stock collection data to inform and optimise the sampling design for surveys that make use of material obtained at the delivery points from fallen stock, plus to identify the potential for bias to guide interpretation of fallen stock acquired diagnostic PM data and so facilitate its utilisation. |
| SRUC Veterinary Services dataset – Stage 3 | |  |  |
| 1 | REC/DIRADC | Improve capture and recording of number of animals included in a submission record | Will facilitate estimation of appropriate unit of analysis and thus accuracy of analysis |
| 2 | REC/DIRADC | Improve recording of number of animals included in a submission record that are allocated/assigned the diagnostic code | Will facilitate estimation of appropriate unit of analysis |
| 3 | REC/DIRADC | Improve capture and recording of ages of animals included in a submission | Will facilitate appropriate analysis, especially where age has a bearing on management, frequency, pathogenesis, or epidemiology of a disease process and subsequent control strategies i.e potential solutions); |
| 4 | REC | Ensure first or repeat submission from an epidemiological unit (e.g. flock/holding). | Facilitate appropriate analysis and more accurate classification of which records to include in specified analyses. |
| 5 | REC/DIRADC | Ensure accurate unique flock/holding identifier recorded for linkage to other data sources. | Will facilitate appropriate analysis, enable value to be added by linkage to other data sources and validation of data already being collected, by comparison. |
| 6 | REC | Agree optimum unit of analysis for use in the specified circumstance/surveillance purpose  e.g. submission-level assigned diagnosis, or animal level; all, or by age categories; here – acute diagnoses only, or chronic too | Facilitate analysis, consistency and interpretation |

DIRADC = Data improvement requiring additional data collection; EDAP = Existing data - additional provision; FIW = Further investigative work; REC = Recommendation

Table SM5: Frequency distribution of the SRUC Veterinary Services ovine diagnostic submission records with VIDA code 372 or 373 during January 2011 to December 2014, inclusive: by primary or secondary diagnosis and by country of origin.

| VIDA Code | Number of diagnostic submission records with this diagnosis | | | |
| --- | --- | --- | --- | --- |
|  | VIDA1 primary diagnosis field | VIDA1  Scottish origin | VIDA1  Non-Scottish origin | VIDA2 secondary diagnosis field (VIDA1 code assigned) |
| 372 | 179 | 178 | 1 | 29 (373) |
| 373 | 769 | 702 | 67 | 10 (372) |
| Other | 88 | 86 | 2 | 15 (372)  73 (373) |

## Supplementary Figures – see supplementary image file

Supplementary Material Figure 1A-F The seasonal patterns for the three age groups of estimated animal units (AUs) by country using the 2011-2018 combined fallen stock datasets.

**
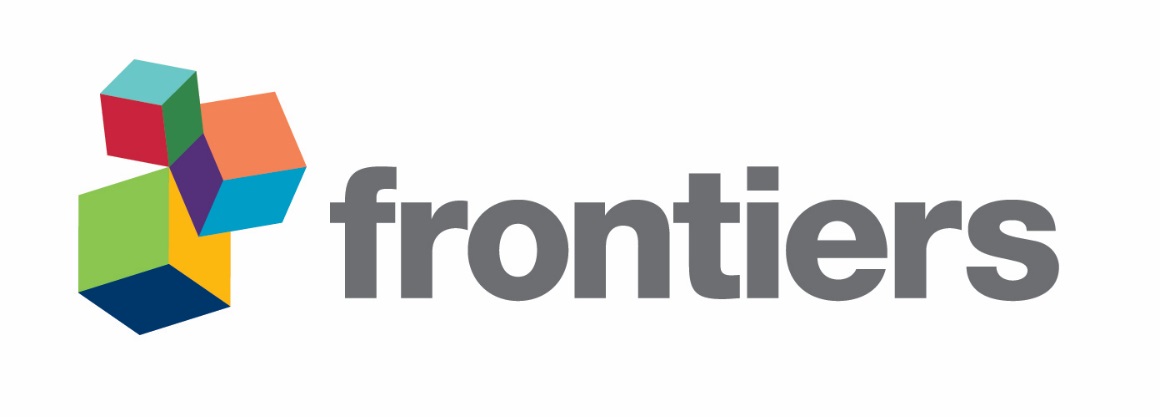
**
