# Supplementary figures and images for: Improving the Utility of Voluntary Ovine Fallen Stock Collection and Laboratory Diagnostic Submission Data for Animal Health Surveillance Purposes: A Development Cycle
Source: Front Vet Sci. 2020 Jan 24;6:487. doi: 10.3389/fvets.2019.00487 (PMC6993589; doi:10.3389/fvets.2019.00487)

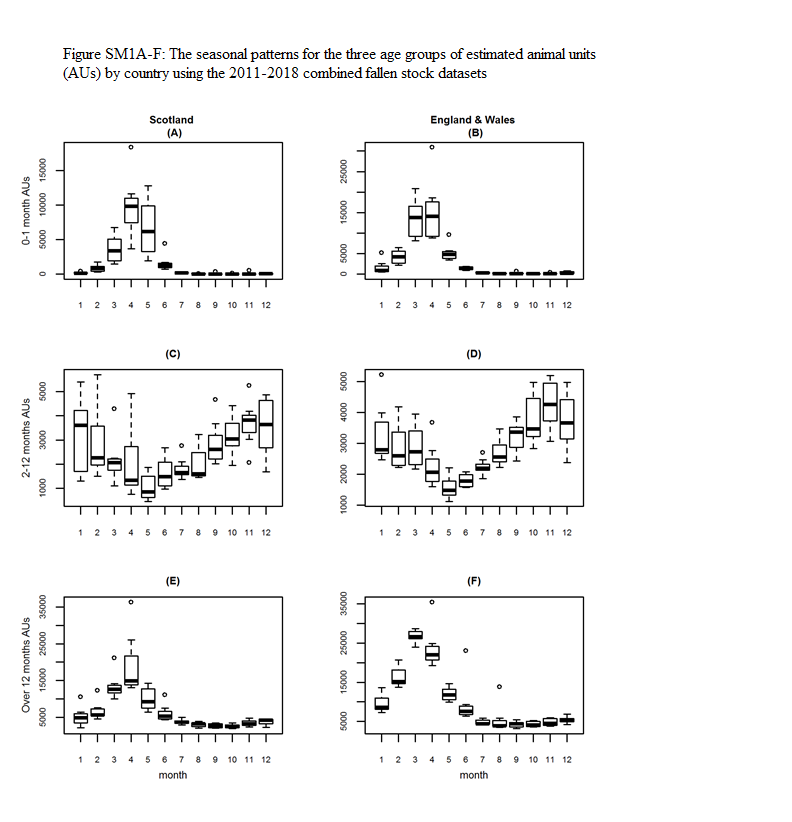

Supplement: Supplementary file 2 [file Image_1.TIF]
